# Favipiravir induces HuNoV viral mutagenesis and infectivity loss with clinical improvement in immunocompromised patients

**DOI:** 10.1016/j.clim.2024.109901

**Published:** 2024-02

**Authors:** Alexandra Y. Kreins, Emma Roux, Juanita Pang, Iek Cheng, Oscar Charles, Sunando Roy, Reem Mohammed, Stephen Owens, David M. Lowe, Rossa Brugha, Rachel Williams, Evey Howley, Timothy Best, E. Graham Davies, Austen Worth, Caroline Solas, Joseph F. Standing, Richard A. Goldstein, Joana Rocha-Pereira, Judith Breuer

**Affiliations:** a Infection, Immunity and Inflammation Research and Teaching Department, Great Ormond Street Institute of Child Health, University College London, London, United Kingdom; b Department of Immunology and Gene Therapy, Great Ormond Street Hospital for Children NHS Foundation Trust, London, United Kingdom; c KU Leuven - Department of Microbiology, Immunology and Transplantation, Rega Institute, Laboratory of Virology and Chemotherapy, Leuven, Belgium; d Department of Pharmacy, Great Ormond Street Hospital for Children NHS Foundation Trust, London, United Kingdom; e Department of Pediatrics, Division of Allergy and Immunology, King Faisal Specialist Hospital & Research Center, Riyadh, Saudi Arabia; f Department of Paediatric Allergy, Immunology and Infectious Diseases, The Newcastle Upon Tyne Hospitals NHS Foundation Trust, Newcastle, United Kingdom; g Immunology Department, Royal Free Hospital NHS Foundation Trust, London, United Kingdom; h Institute of Immunity and Transplantation, University College London, London, UK; i Department of Cardiothoracic Transplantation, Great Ormond Street Hospital for Children NHS Foundation Trust, London, United Kingdom; j Department of Microbiology, Virology and Infection Control, Great Ormond Street Hospital for Children NHS Foundation Trust, London, United Kingdom; k Unité des Virus Émergents IRD 190, INSERM 1207, Aix-Marseille Université, Marseille, France; l APHM, Laboratoire de Pharmacocinétique et Toxicologie, Hôpital La Timone, Marseille, France; m Division of Infection and Immunity, University College London, London, United Kingdom

**Keywords:** Human norovirus infection, Immunodeficiency, Mutagenic antivirals, Favipiravir, Virus whole-genome-sequencing, Zebrafish larvae

## Abstract

Chronic human norovirus (HuNoV) infections in immunocompromised patients result in severe disease, yet approved antivirals are lacking. RNA-dependent RNA polymerase (RdRp) inhibitors inducing viral mutagenesis display broad-spectrum in vitro antiviral activity, but clinical efficacy in HuNoV infections is anecdotal and the potential emergence of drug-resistant variants is concerning. Upon favipiravir (and nitazoxanide) treatment of four immunocompromised patients with life-threatening HuNoV infections, viral whole-genome sequencing showed accumulation of favipiravir-induced mutations which coincided with clinical improvement although treatment failed to clear HuNoV. Infection of zebrafish larvae demonstrated drug-associated loss of viral infectivity and favipiravir treatment showed efficacy despite occurrence of RdRp variants potentially causing favipiravir resistance. This indicates that within-host resistance evolution did not reverse loss of viral fitness caused by genome-wide accumulation of sequence changes. This off-label approach supports the use of mutagenic antivirals for treating prolonged RNA viral infections and further informs the debate surrounding their impact on virus evolution.

## Highlights

• Chronic HuNoV infections can be severe and life-threatening in the immunocompromised.
• Favipiravir-treated patients showed dose-dependent clinical improvement.
• Clinical efficacy derives from genome-wide viral mutagenesis and loss of infectivity.
• No evidence of clinically relevant drug resistance despite intrahost virus evolution.

## 1 Introduction

Highly potent and broadly acting antiviral drugs are in urgent demand, particularly against emerging and re-emerging RNA viruses, including SARS-CoV-2. Inhibitors of the viral RNA-dependent RNA polymerase (RdRp) have great potential to yield broad-spectrum activity, given the high degree of structural and functional RdRp similarity across a wide range of RNA viruses, with the result that many approved anti-RNA virus drugs are nucleoside-based RdRp inhibitors. Some of these drugs appear to act not simply by terminating the incorporation of incoming nucleoside triphosphates into the nascent RNA genome, but rather by inducing errors during this RNA copying process [[1], [2], [3], [4], [5], [6], [7]]. The build-up of these mutations beyond a tolerated error threshold can lead to virus extinction. This process, known as lethal mutagenesis, has been shown to be the main mode of action of ribavirin, favipiravir and more recently molnupiravir, particularly at clinically tolerated drug levels [8].

Although RdRp inhibitors are major candidates when considering antiviral drug repurposing, their clinical use (particularly that of ribavirin and favipiravir) has led to mixed results in the treatment of influenza [[9], [10], [11]], ebola [12,13] and SARS-CoV-2 [6,[14], [15], [16], [17], [18], [19]], as well as human norovirus (HuNoV) infections [20]. Poor clinical responses have been linked to suboptimal pharmacokinetics (namely low drug levels) [21], while modest reductions in viral loads (VLs) have raised doubts as to their anti-viral efficacy [20]. With the rollout of molnupiravir for the treatment of SARS-CoV-2, additional concerns have been raised as to whether the mutagenic mode of action could facilitate the selection of drug-resistant variants and even of new variants of concern [22]. Studies addressing the effect of widespread and/or long-term treatment with mutagenic antivirals are necessary to guide their optimal clinical use in the future, especially regarding the development of drug resistance.

Due to the lack of an approved HuNoV-specific antiviral treatment, several different drugs including ribavirin, nitazoxanide and high-dose immunoglobulins have been repurposed for off-label use as single agents to treat chronic HuNoV infections in immunocompromised patients [23,24]. In contrast to self-limiting infections in immunocompetent hosts, HuNoV infections can be life-threatening in this group of patients who suffer from rare inborn errors of immunity. HuNoV infections in immunodeficient patients are associated with chronic diarrhoea, poor enteral tolerance with frequent dependency on parenteral nutrition and significant weight loss. This disease burden can jeopardise their access to corrective therapies, such as haematopoietic stem cell transplantation (HSCT). While no clinical trials have been conducted in this small cohort of patients, occasional case reports with variable clinical and virological outcomes have been published [[25], [26], [27]]. More recently we reported the use of favipiravir in a patient with common variable immunodeficiency (CVID) and chronic HuNoV infection [20]. We showed clinical improvement associated with the accumulation of mutations in the viral genome during periods of favipiravir treatment given over more than two months.

Here we report the use of favipiravir alone and in combination with nitazoxanide, a drug that has been shown to have broad-spectrum activity against RNA viruses [28] to treat chronic HuNoV infection in three more immunocompromised patients. To provide evidence for drug efficacy or otherwise, we monitored clinical improvement, HuNoV sequence variation in longitudinal samples by means of whole-genome sequencing (WGS), and in one patient, changes in HuNoV infectivity using a small animal model, the zebrafish larvae [29]. The results provide information on the accumulation of favipiravir-driven genome-wide sequence changes in relation to viral fitness and treatment efficacy over time. The data provide evidence to support the use of these regimens as a bridging therapy to improve the health of immunosuppressed patients with chronic HuNoV until immune reconstitution after corrective procedures occurs.

## 2 Materials and methods

### 2.1 Patients and ethics

The Drugs and Therapeutics Committees at Great Ormond Street Hospital for Children, Great North Children's Hospital and Royal Free London NHS Trust approved treatment with favipiravir +/− nitazoxanide in four immunocompromised patients with life-threatening/severe chronic HuNoV infections. After obtaining informed consent, microbiological and pharmacological monitoring was performed. Informed consent was also obtained for microbiological monitoring in 2 untreated patients with HuNoV infections. Residual diagnostic viral samples were released for whole-genome sequencing through the UCL/UCLH Pathogen Biobank National Research Ethics Service Committee London Fulham (reference: 12/LO/1089). For P1 specifically, residual stool samples were released for *in vivo* infectivity evaluation in the zebrafish model with research ethics approval from London Bloomsbury Research Ethics Committee (reference: 07/Q0508/43). This allows the use of pseudonymised residual diagnostic samples for research. An additional stool sample positive for HuNoV from the existing collection of samples of the University Hospital of Leuven (Belgium) was used (reference: G-2021–4376). Informed consent was obtained from the patient. All zebrafish experiments were approved and performed according to the rules and regulations of the Ethical Committee of KU Leuven (reference: P086/2017), in compliance with the regulations of the European Union (EU) concerning the welfare of laboratory animals as declared in Directive 2010/63/EU. Zebrafish larvae were used from 72 h post fertilization (hpf) until a maximum of 144 hpf.

### 2.2 Drug levels and pharmacokinetic modelling

Plasma was isolated from patient blood samples collected in EDTA. Frozen plasma samples were shipped to Laboratoire de Pharmacocinétique-Toxicologie, Hôpital La Timone, Aix-Marseille Université, Marseille, France for favipiravir concentration analysis. The quantification of favipiravir in thawed plasma samples was performed by ultra-performance liquid chromatography coupled with tandem mass spectrometry (UPLC-MS/MS) method (UPLC-TQD, Waters, USA) with a lower limit of quantification of 0.5 μg/mL [34]. Validation of the assay was performed in accordance with the 2012 EMA guidelines and the ISO15189 guidelines. Model parameter estimation, model simulation properties (visual predictive check) and dose simulations were assessed using nlmixr2 (2.0.6) and rxode2 (2.0.7) in R (4.2.0), as described elsewhere [36].

### 2.3 Whole genome sequencing and analysis

Stool samples were collected from the patients. Full-length HuNoV genome sequences were obtained from samples using SureSelectXT target enrichment and Illumina sequencing. For each patient, a unique patient reference was generated by mapping the remaining reads of the first sample to the genotype reference from GenBank using bwa-mem [57]. Reads from the subsequent samples of the same patient were mapped to this patient reference. Consensus sequences were aligned using MAFFT [58]. Only genomes with >80% genome coverage and a mean read depth of 100 or above were included in downstream analysis. Minority allele variants with a frequency of above 2% and a minimum of 2 supporting reads were identified at sites with a read depth of ≥5 using VarScan [59]. For plotting polymorphism frequency, we have determined Watterson's theta which normalises for reading depth. Maximum likelihood phylogenies were constructed using RAxML [13], with the GTR model and 1000 bootstrap replicates. General data processing was carried out in R 3.6.1 using Rstudio 1.2. with the tidyverse family of packages (v1.2.1). Figures were made using the ggplot2 package.

### 2.4 Structural biology

To visualise the distribution and context of RdRp AA changes occurring under treatment we generated a protein structure model of the Patient 1 t0 consensus using Alphafold (v.2.1.1) [60]. This had the advantage of including all residues and excellent homology to the consensus sequences used in this analysis, with >98% minimum identity. The required databases were downloaded on 2 December 2021 and the program was run with the parameters –max_template_date = 2021-12-01 – model_preset = monomer –db_preset = full. The resulting model showed good alignment with PDB entry 1SH0 with a root mean square distance of 0.49 Å between atom pairs. The finger-palm-thumb structures of the RdRp were inferred from homology to the SARS-CoV-2 RdRp fingers (398–581,628–607), palm (688–815, 582–627) and thumb (816–932) structures [61]. To assess the impact of the A44S mutant, the catalytic domain of 1SH0 was visualised, then alanine was substituted for serine with the optimal amino acid rotamer chosen by optimal prevalence using the Dunbrack rotamer library [62]. Possible novel hydrogen bonding interactions were then identified to S44 without further structure relaxation, using constraints of 0.4 Å and 20 degrees of rotational freedom.

### 2.5 Zebrafish maintenance

Wild type AB adult zebrafish were maintained in the aquatic facility of the KU Leuven (temperature of 28 °C and 14/10 h light/dark cycle). Fertilized eggs were collected from adults placed in mating cages and kept in petri dishes containing Danieau's solution (1.5 mM HEPES, 17.4 mM NaCl, 0.21 mM KCl, 0.12 mM MgSO_4_, 0.18 mM Ca(NO_3_)_2_ and 0.6 μM methylene blue) at 28 °C until the start of experiments.

### 2.6 Collection and processing of HuNoV positive stool samples

Human stool samples, positive for HuNoV, were obtained from the Great Ormond Street Hospital for Children (United Kingdom) and the University Hospital of Leuven (Belgium). An aliquot of 100 mg of each stool sample was re-suspended in 1 mL of sterile PBS, thoroughly vortexed, and centrifuged (5 min, 1000 *g*), supernatant was harvested and stored at −80 °C. This virus suspension was used for RNA extractions, quantification by RT-qPCR and injections in the zebrafish larvae. HuNoV RNA was extracted from 100 μL of PBS suspension using the Direct-zol RNA kit (Zymo Research, Irvine, CA, USA), according to the manufacturer's protocol. The original concentrations of the stool samples were as followed: SS A: 5.52*10^9^ copies/g stool, SS B: 1.34*10^11^ copies/g stool, SS C: 2.07*10^11^ copies/g stool, SS D: 3.07*10^10^ copies/g stool, SS E: 1.23*10^12^ copies/g stool, SS F: 3.12*10^11^ copies/g stool and GII.4 Sydney[P16] (*in house* collection KU Leuven): 1.46*10^10^ copies/g stool.

### 2.7 Antiviral treatment of the zebrafish larvae

First, compounds were dissolved in 100% DMSO (spectroscopy grade). To determine the correct dose for pericardial injections in zebrafish larvae of all compounds (i.e. favipiravir, tizoxanide and their 1:1 combination), a weight-dependent conversion was applied. For this purpose, the optimized and increased clinical dose of 400 mg favipiravir TDS and 200 mg tizoxanide BD was used. To ensure the weight-converted dose was not toxic for zebrafish larvae of 3 days post fertilization, a toxicity analysis was performed [41]. The maximum tolerated dose of the test conditions was determined as the dose at which no larvae died, nor showed signs of toxicity or locomotor impairment in comparison to DMSO-treated control larvae after a period of 18 h post-treatment. The following parameters were investigated: touch response, morphology, posture, oedema, signs of necrosis, swim bladder and heartbeat.

### 2.8 Injections of virus and antiviral treatment in zebrafish larvae

Zebrafish larvae were anesthetized and positioned as described previously [29,41,63], in short: zebrafish larvae of 3 days post fertilization were anesthetized and transferred to an agarose mold to position them on their dorsal side with the yolk facing upward. In every experiment, the injection needle was calibrated to ensure the precision of the injection volume. Microinjection was performed using an M3301R Manual Micromanipulator (WPI, Friedberg, Germany) and a FemtoJet 4i pressure microinjector (Eppendorf, Hamburg, Germany). Compound-treated zebrafish larvae received a 1 nL (1.5 nL in case of the combination treatment) injection of compound in the pericardial cavity, while negative control zebrafish were injected with an equal volume of DMSO. Subsequently, the zebrafish larvae received an injection of 3 nL virus in the yolk. After both injections, zebrafish larvae were transferred to 6-well plates with Danieau's solution and further maintained in an incubator with a 14/10 h light/dark cycle at 32 °C. Each day post injection, the general condition of the zebrafish larvae (e.g. posture, swimming behaviour or signs of oedema) was observed to record clinical signs of virus infection and antiviral treatment, and 10 zebrafish larvae were collected into 2 mL tubes containing 2.8 mm zirconium oxide beads (Precellys/Bertin Technologies, Montigny-le-Bretonneux, France) and stored at −80 °C until further processing.

### 2.9 Tissue homogenization, RNA extraction, and RT-qPCR for quantification of human HuNoV

Zebrafish larvae were homogenized (Precellys 24, Bertin Technologies, Montigny-le-Bretonneux, France), the homogenates were cleared by centrifugation (5 min, 9000 *g*), and RNA was extracted using the Direct-zol RNA kit (Zymo Research, Irvine, CA, USA), according to the manufacturer's protocol. RNA levels were quantified with a one-step RT-qPCR (iTaq Universal Probes One-Step Kit, Bio-Rad, Hercules, CA, USA) as previously described [29,41,63].

### 2.10 Viral infectivity calculation

To start, all stool samples were brought to the same theoretical inoculum (± 1000 viral RNA copies/3 nL injection volume) by dilution with PBS. To compare the viral infectivity between different stool samples, multiple methods were used; A) calculating the fold increase by taking the input based on the viral load of P1's stool samples and the amount of viral RNA copies/zebrafish at the peak of replication into account which are both quantified by RT-qPCR and B) determining the 50% infectious dose for each stool sample based on a well-established method [64] adapted to the zebrafish replication model by diluting the collected stool samples to evaluate at which dilution the infectivity is lost.

### 2.11 Characterization of the immune response by *IRF-1* and *Mx* upon treatment with tizoxanide

To generate the cDNA, the ImProm-II Reverse Transcription System (Promega, Madison, WI, USA) was used. Briefly, a total of 1 μg (ca. 8 μL) of extracted RNA was added to 2 μL of random hexamers and incubated at 70 °C for 5 min, followed by 5 min at 4 °C. To this reaction mix a total volume of 30 μL containing 8 μL of Improm II 5× reaction buffer, 6 mM MgCl_2_, 0.5 mM deoxynucleoside triphosphate, 40 units of RNase inhibitor, 2 μL of Improm II reverse transcriptase, followed by an incubation at 25 °C for 5 min, 37 °C for 1 h, and 72 °C for 15 min. A qPCR was performed with 4 μL template cDNA using the SsoAdvanced Universal SYBR green supermix, 600 nM of forward and reverse primers for *IRF-1*, *Mx* and the housekeeping genes *18 s*, *β-actin*, and *ef1a*. Primer sequences were as previously described [[65], [66], [67], [68]]. Cycling conditions were as follows; polymerase activation at 95 °C for 3 min followed by 40 cycles of denaturation at 95 °C for 15 s, annealing at 55 °C and extension at 72 °C for 30 s (Quantstudio 5, ThermoFisher Scientific, Waltham, MA, USA). Data was normalized to the housekeeping genes and compared to DMSO-injected zebrafish larvae infected with HuNoV to determine the fold induction of the expression, according to the Livak method [69].

### 2.12 Statistics

Data was analysed using GraphPad Prism 9.12 (GraphPad Software, San Diego, CA, USA) and *p* values were determined using the Mann-Whitney *U* test (Fig. 2.A-D and Fig. 4.D) and the one sample *t-*test compared to the theoretical value 1 (Fig. 2.E), where **** *p* < 0.0001, *** *p* < 0.001, ** *p* < 0.01, * *p* < 0.05, and ns is *p* ≥ 0.05. Outlier test (ROUT, Q = 1%) was performed to identify and exclude potential outliers (Fig. 2.A-B/E).

## 3 Results

### 3.1 Clinical response to favipiravir in immunodeficient patients with chronic HuNoV infection is dose- dependent

**Fig. 1:**
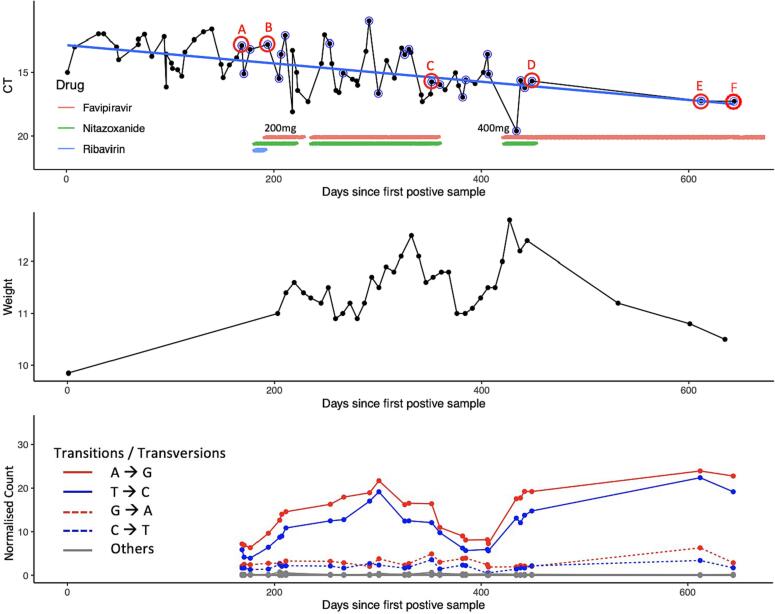
Clinical improvement is associated with accumulation of favipiravir-induced viral sequence changes: Monitoring of treatment response in patient P1 over time, indicated in days since first positive stool sample. Treatment periods are indicated at the top (red line for favipiravir, green line for nitazoxanide, blue line for ribavirin); dose of favipiravir is specified. Samples which have been processed for viral deep sequencing are circled in blue. Stool samples outlined in red (A-F), were used for in vivo experiments in zebrafish larvae. The top panel shows HuNoV viral load quantification by RT- qPCR from stool samples obtained during the same period. The viral load is expressed in cycle threshold (Ct) values, with increasing Ct values corresponding to a reduction in viral load. The middle panel shows patient weight before and during antiviral treatment, as one of the clinical parameters used to monitor treatment outcomes. The bottom panel shows the counts of variants A → G and T → C; solid lines, associated with favipiravir mutagenesis and G → A and C → T dotted lines, complementary strand mutations for each stool sample processed for viral deep sequencing before and during the periods of favipiravir treatment, normalized using Watterson's theta. Other transitions and transversions are shown in grey. Results for the samples used in the in vivo experiments are again indicated by A-F (in red). (For interpretation of the references to colour in this figure legend, the reader is referred to the web version of this article.)

Six immunocompromised patients (P1-P6) were diagnosed with chronic infection due to HuNoV. Their clinical details are summarised in Table 1. In brief, three patients with inborn errors of immunity (IEI), P1-P3, developed severe enteropathy with chronic diarrhoea and failure to thrive (FTT), making them ineligible for corrective cellular treatment procedures. A fourth patient, P4, under long-term immunosuppressive treatment after a bilateral lung transplant, also developed chronic enteropathy with persistent feeding difficulties and diarrhoea. For this study antiviral treatment was initiated with favipiravir alone (P2 and P3) [20] or together with nitazoxanide (P1 and P4). Two chronically HuNoV-infected patients, P5 and P6, did not receive any antiviral treatment. Informed consent was obtained for longitudinal collection of stool samples for virological monitoring before and where applicable, during treatment with favipiravir, in all six patients. Repeated PCR analysis of their stool revealed extremely high HuNoV viral loads (VLs) (Fig. 1 and Supplementary Fig. 1.A-E).

**Table 1:** Clinical summary P1-P6:

PATIENT	DIAGNOSIS	HuNoV INFECTION	ANTIVIRAL TREATMENT	CLINICAL RESPONSE
P1 (5yo)	**Athymic SCID** Post-(failed) HSCT Pre-thymus transplantation	**Chronic enteropathy** **FTT, diarrhoea, PN**	Nitazoxanide, ribavirin **Nitazoxanide, favipiravir** **Favipiravir**	**Weight gain, reduced stool output, improved enteral feeding**
P2 (48yo)	**CVID** Pre-HSCT	**Chronic enteropathy** **FTT, diarrhoea, PN**	**Favipiravir**	**Weight gain, reduced stool output, decreased adjunctive loperamide**
P3 (12yo)	**CID** Pre-HSCT	**Chronic enteropathy** **FTT, diarrhoea, NG feeds**	**Favipiravir**	**Weight gain, reduced stool output, improved enteral feeding**
P4 (6yo)	**Post-bilateral lung transplant**	**Chronic enteropathy** **FTT, emesis, diarrhoea**	**Nitazoxanide, favipiravir**	**Weight gain, reduced stool output**
P5 (1yo)	**Histiocytic sarcoma** Pre-HSCT	Asymptomatic	None	NA
P6 (1yo)	**Wiskott Aldrich Syndome** Pre-HSCT	Acute gastroenteritis	None	NA

P1, a 5-year-old IEI patient with severe combined immunodeficiency (SCID) [30], was admitted to our center for consideration of thymus transplantation [31]. She had FTT with a weight of 11 kg and a history of chronic enteritis. She was diagnosed with norovirus enteropathy with very high stool HuNoV VLs (with cycle thresholds (Cts) between 11.6 and 16.2), associated with severe diarrhoea (type 7 by the Bristol stool scale), malabsorption and weight loss, requiring parenteral nutrition (PN). We started treatment with 12-hourly ribavirin 84 mg in combination with 12-hourly nitazoxanide 200 mg. Due to its known toxicity [32], ribavirin was replaced 10 days later with 8-hourly favipiravir 200 mg (51 mg/kg/day) upon approval for its use. In the first three months following the start of treatment, P1 gained sufficient weight (Fig. 1) and muscle to proceed with thymus transplantation, which requires tissue implantation into the quadriceps muscle [33]. Despite improvements, the patient remained PN- dependent, with no change in enteral feeding, stool output and VLs (Fig. 1). Because recovery of T- cell immunity after thymus transplantation typically takes months and active viral infections can drive life-threatening inflammatory complications during immune reconstitution [33], antiviral treatment was continued after thymus transplantation. In absence of paediatric PK data, plasma samples were collected for drug level monitoring [34]. The average plasma trough level measured at month four (10.0 μg/mL) was below the reported EC_50_ for mouse norovirus in vitro (39 μg/mL with 95%CI: 35–43 μg/mL) [35] (Table 2); no EC_50_ values are available for HuNoV. In-house pharmacokinetic modelling [36] predicted a median steady state plasma concentration (Css) of 34.7 μg/mL (95% CI: 14.5–77.7 μg/mL) (Table 2). At this time, the patient became critically ill and developed conjugated hyperbilirubinaemia, which was suspected to be drug-induced. Several medications, including nitazoxanide and favipiravir, were discontinued. In the face of continued PN-dependence and diarrhoea, once the hyperbilirubinaemia had recovered, the patient was restarted on 12-hourly nitazoxanide 200 mg and an increased dose of favipiravir 400 mg 8-hourly (97 mg/kg/day), resulting in higher favipiravir blood levels (plasma trough level: 154 μg/mL; predicted median Css: 69.4 μg/mL with 95%CI: 29.0–155.5 μg/mL) (Table 2). On the higher dose of favipiravir, we observed rapid and significant reduction of the disease burden with improved enteral feeding, documented decreased stool output, more formed stools and progressive weight gain (Fig. 1). HuNoV VLs showed only a modest reduction with Cts between 15.6 and 17.3 (Fig. 1). The conjugated hyperbilirubinaemia did not recur. PN was successfully weaned and P1 was discharged home, tolerating enteral feeds. Due to supply issues, nitazoxanide was discontinued nine months after initial administration and favipiravir monotherapy was continued. P1 remained well with reduced diarrhoea and tolerating enteral feeds. After 3 months of monotherapy, P1 developed central line-associated sepsis and died one year after thymus transplantation, before achieving immune reconstitution [37].

**Table 2:** Measured and predicted favipiravir plasma concentrations in P1–4:

PATIENT	DOSE REGIMEN	TROUGH LEVEL (μg/mL)	PREDICTED MEAN CSS (μg/mL)
P1	200 mg TDS	10.0	34.7 (14.5; 77.7)
P1	400 mg TDS	154.0	69.4 (29.0; 155.5)
P2	1200 mg BD	NA	31.2 (27.9; 35.2)
P3	1200 mg TDS	NA	52.5 (42.5; 67.3)
P3	800 mg BD	NA	35.0 (28.3; 44.9)
P4	400 mg TDS	159.0	32.4 (23.3; 50.2)

EC_50_ Favipiravir for mouse norovirus (39 ± μg/mL [35])

Css steady-state plasma concentration.

P2, a 48-year-old CVID patient with deteriorating chronic enteropathy, who has been previously reported [20], was treated with three courses of 12-hourly favipiravir 1200 mg (for 19 days, 6 days and 41 days), each leading to weight gain, improvement in stool output and a reduction in the use of anti-diarrhoeal drugs. These changes were associated with a modest decrease in HuNoV VLs, but not viral clearance (Supplementary Fig. 1.A). With this dose regimen, a median Css of 31.2 μg/mL is predicted (95% CI: 27.9–35.2 μg/mL) (Table 2). Treatment interruptions were due to recurring transaminitis of undetermined aetiology and were associated with increases in VLs and relapsing gastro-intestinal symptoms. The patient, who was also diagnosed with *Mycobacterium avium*- associated bronchiolitis, died from respiratory failure unrelated to his norovirus infection [30].

P3, a 12-year-old Combined Immunodeficiency (CID) patient requiring nasogastric feeding with a weight of 28.9 kg and high stool HuNoV VLs (max Ct of 21.2), received favipiravir monotherapy 12- hourly 600 mg increasing to 12-hourly 1200 mg (83 mg/kg/day) after one week, with a predicted median Css of 52.5 μg/mL (95% CI: 42.5–67.3 μg/mL) (Table 2). HuNoV VLs rapidly decreased (Ct of 30.9) (Supplementary Fig. 1.B), but due to progressing transaminitis, treatment was discontinued after one month. Within one week of favipiravir cessation, VLs increased to pre-treatment levels with unchanged stool output and FTT. One month later, treatment was reintroduced at the lower dose of 12-hourly favipiravir 400 mg and progressively increased to 12-hourly 800 mg (50 mg/kg/day), with predicted median Css of 35.0 μg/mL (95% CI: 28.3–44.9 μg/mL) (Table 2). Transaminitis did not recur, but P3 developed nail discolouration and frequent headaches. Over a treatment period of four months, P3's weight increased to 33.3 kg (Supplementary Fig. 1.B), stool output decreased from 6 to 10 to 2–4 stools per day, and nasogastric feeds were successfully weaned. No change in stool HuNoV VLs was observed (Cts between 24.0 and 27.8) (Supplementary Fig. 1.B), but the remarkable weight gain and reduction in disease burden following the start of antiviral treatment made the referral for HSCT possible. P3 cleared the HuNoV infection upon achieving full donor T-cell engraftment and good immune reconstitution.

P4, a 6-year-old patient with secondary immunodeficiency due to immunosuppression after lung transplantation, a weight of 17.9 kg (13^th^ centile) and high HuNoV VL (max Ct of 17.9) (Supplementary Fig. 1.C), was treated with 8-hourly favipiravir 400 mg (67 mg/kg/day) and 12-hourly nitazoxanide 200 mg. We confirmed good favipiravir blood levels (plasma trough level: 159 μg/mL; predicted median Css: 32.4 μg/mL with 95%CI: 23.3–50.2 μg/mL) (Table 2) associated with decreasing HuNoV VLs (max Ct of 27.8) and clinical improvement, including reduced stool output and weight gain (34th centile) (Supplementary Fig. 1.C). After 2 months of treatment, P4 developed nail discolouration and intermittent jaundice. Liver function could not be monitored regularly but was not altered when assessed. Following discontinuation of treatment, the nail discolouration reversed. The medical team elected not to reintroduce treatment at a lower dose, because close clinical and laboratory monitoring could not easily be arranged. Gastrointestinal symptoms subsequently worsened resulting in weight loss (14^th^ centile), while HuNoV VL increased again to high levels which was associated with return of symptomatic diarrhoea and weight loss (Supplementary Fig. 1.C).

In summary, P1-P4 all showed clinical improvement in response to favipiravir treatment in absence of functional T-cell immunity with deterioration of symptoms on treatment interruption. As seen in P1, P2 and P3, treatment responses were dose-dependent.

### 3.2 Viral deep sequencing confirms favipiravir-induced mutation bias

In the absence of T-cell immunity, viral clearance or at least significant VL reductions may not be achievable due to the need for achieving concentrations well above EC_50_ [38], which may result in toxicity, as occurred in P3 at high-dose favipiravir treatment. Nevertheless, better tolerated lower favipiravir doses, in combination with nitazoxanide (in P1 and P4) or alone (in P2 and P3), also led to clinical improvement despite modest reductions in VLs (Fig. 1 and Supplementary Fig. 1), possibly indicating reduced viral pathogenicity under treatment. We therefore performed whole-genome- sequencing (WGS) on HuNoV [39] from longitudinally collected stool samples to determine whether drug-associated changes in HuNoV sequences correlated with the apparent clinical improvement.

For P1, a total of 25 samples were sequenced (Fig. 1), including four samples collected before starting treatment, 10 samples during the first favipiravir treatment course (at 8-hourly favipiravir 200 mg) and 6 during the second treatment course (at 8-hourly favipiravir 400 mg), making it possible to study treatment outcomes in detail. Viral genotyping (Supplementary Fig. 2.A-D) revealed P1 and P3 virus to be the currently predominant GII.4 Sydney[P16] 2012 HuNoV strain (Supplementary Fig. 2.A and C). P2 virus was closer to GII.4 strains circulating prior to 2006 (Supplementary Fig. 2.B), as has previously been reported [20], and P4 was infected with a GII.2 strain (Supplementary Fig. 2.D). None of the patients showed evidence of mixed genotypes or re-infection (Supplementary Fig. 2). In line with previous studies showing favipiravir-induced mutagenesis [4,20,40], including the previous report on P2 [20] (see also Supplementary Fig. 1.A), we observed accumulation of A to G and T to C sequence changes in subjects P1, P2 and P4, coinciding with periods of favipiravir treatment, and reducing in frequency after treatment cessation (Fig. 1 and Supplementary Fig. 1.A and C). This accumulation of favipiravir-associated sequence changes is particularly evident over time for P1, who was treated over a longer period and in whom more samples have been sequenced (Fig. 1). Viral deep sequencing was also performed on six samples from P3 (three collected during the initial treatment course, two during treatment interruption and one at the beginning of the second treatment course) (Supplementary Fig. 1.B). This revealed a single genotype with new non- synonymous (NS) changes occurring over time, yet without evidence of accumulation of A to G and T to C favipiravir-associated sequence changes (Supplementary Fig. 1.B). No samples were available for sequencing from the later stages of the second treatment course using the lower favipiravir dose regimen.

Next, we compared these findings with those of samples collected over a period of 2 and 3 months respectively from P5 (6 samples) and P6 (7 samples), who did not receive favipiravir or other antiviral treatment. Genome sequencing of the samples from untreated patients showed P5 to be infected with genotype GII·P7_GII.6 and P6 with genotype GII·P2_GII.2 (Supplementary Fig. 2.E-F). VLs, frequency and nature of low-level and consensus polymorphisms over time for these two untreated patients are shown in Supplementary Fig. 1.D-E. We observe that the numbers of sequence changes in these untreated patients (P5 and P6) were significantly lower than for the favipiravir-treated patients P1–4 (*p* = 2.25 × 10^−6^, Student's *t*-test) and that the proportion of low-level polymorphisms due to A to G and T to C changes were also lower than in P1, P2 and P4 (P3 did not have low level favipiravir- associated sequence changes).

Together these results confirm a favipiravir-induced increase in specific sequence changes, which, although associated with drug levels below those required to achieve successful viral clearance, coincided with clinical improvement.

### 3.3 Favipiravir and nitazoxanide both inhibit HuNoV replication in zebrafish larval model

Studies on the efficacy of antivirals against HuNoV were not possible until recently, due to a historic lack of in vitro and in vivo replication models. We recently established a robust small animal model for HuNoV using zebrafish larvae, a model that allows efficient viral replication in a manner that recapitulates infection of the human host [29].

**Fig. 2:**
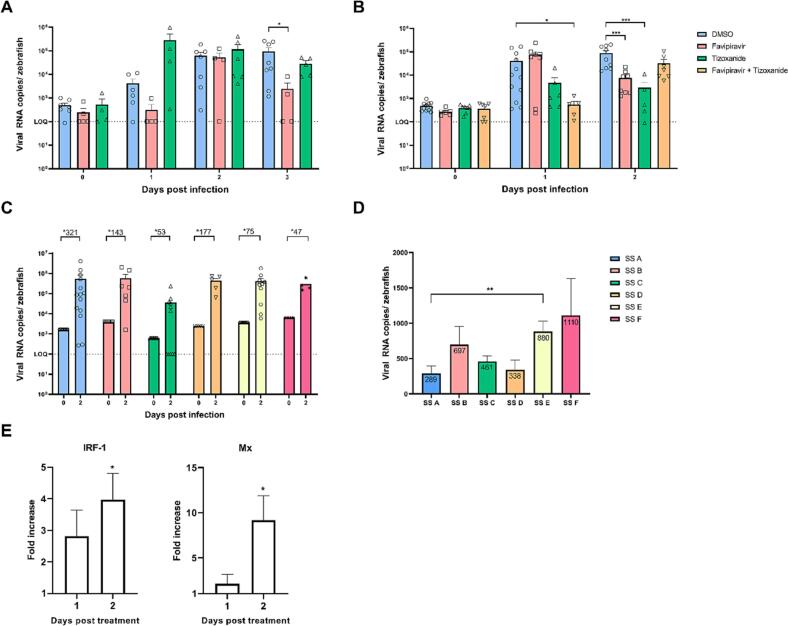
Favipiravir and tizoxanide inhibit HuNoV replication in the zebrafish larval model: A. Favipiravir inhibits HuNoV GII.4 Sydney[P16] replication in zebrafish larvae at the peak of replication (i.e. 3 dpi) using the pre-treatment SS A for infections. B. Both favipiravir and tizoxanide inhibit HuNoV GII.4 Sydney[P16] replication in zebrafish larvae at the peak of replication (i.e. 2 dpi) using a stool sample of the same genotype from the *in-house* collection. For both graphs in 2.A-B: Bars represent viral RNA copies/zebrafish, quantified by RT-qPCR. The dotted line represents the limit of quantification (LOQ). In every independent experiment (*n* = 4–12), 10 zebrafish larvae were harvested at each time point. Mean values ± SEM are presented, outliers were removed (ROUT-test, Q = 1%), Mann-Whitney *U* test, where **** *p* < 0.0001, *** *p* < 0.001, ** *p* < 0.01, * *p* < 0.05. C. HuNoV loses fitness and infectivity upon treatment with favipiravir. Bars represent viral RNA copies/zebrafish, quantified by RT-qPCR and are shown together with the fold increase in viral RNA copy numbers from 0 to 2 dpi. The dotted line represents the limit of quantification (LOQ). In every independent experiment (n = 4–14), 10 zebrafish larvae were harvested at each time point. Mean values ± SEM are presented. D. Bars represent the ID_50_ or the number of viral RNA copies per zebrafish needed to achieve a successful infection in 50% of the cases. Mean values ± SEM are presented, Mann-Whitney U test, where ** *p* < 0.01. E. Antiviral treatment with tizoxanide enhances the expression of innate immune genes *IRF-1* and *Mx*. Bars represent the fold increase in expression of the immune genes *IRF-1* and *Mx* in HuNoV-infected zebrafish larvae treated with tizoxanide compared to HuNoV-infected zebrafish larvae treated with DMSO, as determined by RT-qPCR and normalized to the housekeeping genes. In every independent experiment (*n* = 5), 10 zebrafish larvae were harvested at each time point. Mean values ± SEM are presented, one sample *t*-test compared to a value of 1, where * p < 0.05.

We first evaluated the in vivo antiviral activity of favipiravir and tizoxanide (the active metabolite of nitazoxanide) by injecting each compound into the pericardial cavity of 3-day old larvae, immediately before virus inoculation through the yolk [41] using a stool sample obtained from P1 that was collected on day 193 (from start of monitoring) and before the start of treatment. This sample is henceforth designated stool sample (SS) A as indicated in Fig. 1.A. We determined the drug doses used in the zebrafish, 25 ng of favipiravir and 4 ng tizoxanide, based on weight-dependent conversions of doses used in P1 (i.e. 8-hourly treatment with 400 mg favipiravir and 12-hourly treatment with 200 mg nitazoxanide), using a previously reported method [41]. These doses, when the drug was administered alone, did not show toxicity in the zebrafish larval model. A single dose injection of favipiravir alone resulted in a 1.6 log_10_ reduction in viral RNA copies/zebrafish (*p* = 0.0263) at the peak of replication (i.e. day 3 post infection (pi)) (Fig. 2.A). Treatment with tizoxanide alone had no significant effect (*p* > 0.05) on HuNoV replication when using SS A as inoculum (Fig. 2.A). We next used a higher tizoxanide dose (6 ng), but the larvae showed signs of toxicity, including heart oedema, necrosis and spine curvature. To evaluate the combination of favipiravir and nitazoxanide, we used another wild type HuNoV with the same capsid and polymerase as the patient SS A (GII.4 Sydney[P16]). Favipiravir (25 ng), and tizoxanide (4 ng) monotherapy reduced HuNoV replication by >1 log_10_ (*p* = 0.0006 and 0.001, respectively) (Fig. 2.B). Combination treatment at these doses caused increased mortality rate, oedema, necrosis and spine curvature. As a result, the doses of each compound in the combination treatment were decreased to 19 ng favipiravir and 3 ng tizoxanide. At day 1 pi, combination treatment reduced HuNoV replication in the zebrafish larval model by 1.9 log_10_ (*p* = 0.0136) (Fig. 2.B). At the peak of replication (i.e. day 2 pi), this antiviral effect was less pronounced (p > 0.05).

### 3.4 Favipiravir decreases HuNoV infectivity in the zebrafish larval model

We next evaluated whether treatment with favipiravir led to a loss of HuNoV infectivity. Zebrafish larvae were injected with stool samples (SS A to F) from P1 collected at multiple time points prior to and during treatment (Fig. 1.A). When an inoculum of ∼1000 genome copies of each sample was injected into each larva, it resulted in a fold increase of 321 ± 177 in viral RNA at day 2 pi for SS A (pre- treatment), while SS B and C (collected 2 and 125 days into favipiravir treatment; P1 had previously received 10 days of treatment with ribavirin) yielded only a fold increase of 143 ± 76 and 53 ± 31 in viral RNA, respectively (Fig. 2.C). After a treatment gap of 2 months, HuNoV infectivity (SS D) increased with a fold increase of 177 ± 55 in viral RNA copies at the peak of replication. This declined once again to a fold increase of 75 ± 21 (SS E) and 47 ± 16 (SS F) respectively 6 and 7 months after treatment re-introduction using a higher dose of favipiravir (including several months as monotherapy) (Fig. 2.C). We estimated the 50% infectious dose (ID_50_) by inoculating larvae with serial dilutions of SS A to F (Supplementary Fig. 3). The ID_50_ of the pre-treatment sample (SS A) was calculated as 289 viral RNA copies/zebrafish, which increases to 697 (SS B) and 461 (SS C) viral RNA copies/zebrafish after 2 and 125 days into treatment (Supplementary Fig. 3.A-C), thus confirming decreased infectivity (Fig. 2.D). After a 2-month treatment gap, viral infectivity increased with the ID_50_ falling to 338 viral RNA copies/zebrafish (SS D) (Supplementary Fig. 3.D). Treatment reintroduction again reduced HuNoV infectivity (Fig. 2.D) with the ID_50_ rising to 880 viral RNA copies/zebrafish for SS E and to 1110 viral RNA copies/zebrafish for SS F (Supplementary Fig. 3.E-F). Taken together, samples obtained during periods of treatment showed between 1.2- and 3.8-fold decrease in infectivity compared with the untreated sample A. ID_50_ seemed to show an increase with favipiravir polymorphism count (*r* = 0.71), although this was not significant (*p* = 0.0681) (Supplementary Fig. 3.G).

### 3.5 Nitazoxanide stimulates the innate immune response which limits HuNoV replication in vivo

Given drug toxicity when using higher doses of favipiravir, combination treatment with other antivirals such as nitazoxanide has been considered (as for P1 and P4). Although the in vivo mechanism of nitazoxanide action against HuNoV remains unclear [42,43], its broad-spectrum antiviral activity against other RNA viruses has been linked to boosting of the innate immune response [[44], [45], [46]]. For example, nitazoxanide and its active metabolite tizoxanide increase expression of interferon- stimulated genes in Huh-7 cells, Caco-2 cells and human intestinal organoids [47]. To further investigate this using the zebrafish larvae model, we analysed the expression of innate immune genes *IRF-1* and its downstream signaling factor *Mx* in zebrafish larvae infected with HuNoV (GII.4 Sydney[P16]) and treated with 4 ng tizoxanide. Results were compared with zebrafish larvae of the same age, infected with the same viral inoculum but without tizoxanide. At day 2 pi, the expression of *IRF-1* and *Mx* after tizoxanide treatment were increased 4- (*p* = 0.023) and 9-fold (*p* = 0.0403) respectively compared to untreated larvae (Fig. 2.E). These results indicate boosting of the innate immune response by tizoxanide treatment in infected zebrafish larvae, confirming its potential to contribute to the inhibition of HuNoV replication in vivo*.*

In summary, upon treatment of infected zebrafish larvae, we confirm that favipiravir and tizoxanide reduce HuNoV replication. Moreover, we for the first time show an association between clinical improvement and loss of HuNoV infectivity further supporting drug efficacy.

### 3.6 Emergence of putatively resistant clones with RdRp sequence changes during treatment

**Fig. 3:**
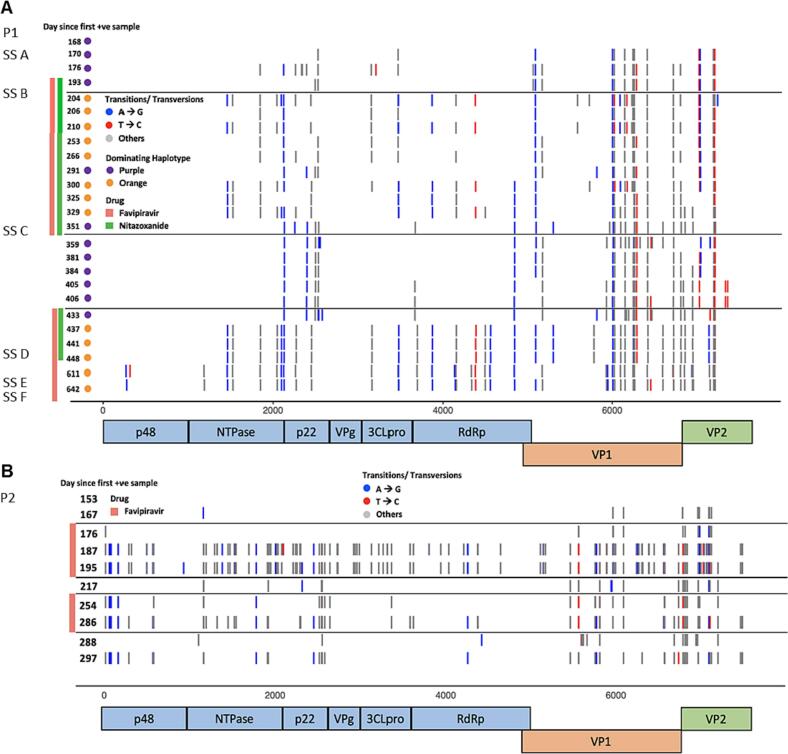
HuNoV RdRp sequence changes occur during prolonged treatment: A-B. Waterfall plots for P1 (A) and P2 (B) showing all non-synonymous (NS) changes at consensus level (>50%) in each sample collected and sequenced over time compared to the baseline patient reference sample. X-axis = positions across the norovirus genome, Y-axis (each row) = each sample. Vertical bars on the left indicate whether the sample was taken during treatment (red = favipiravir, green = nitazoxanide). Sequence changes likely to be related to favipiravir treatment are shown in blue (A to G) and red (T to C). Consensus changes due to other nucleotide transitions and transversions are shown in grey. For P1, the samples used in the zebrafish experiments are labeled SS A-F. (For interpretation of the references to colour in this figure legend, the reader is referred to the web version of this article.)

In addition to uncertainty regarding efficacy, concerns have been raised that, over time, mutagenic antiviral agents could give rise to drug resistant viral clones or favour the selection of viable immune escape variants. To examine this, we analysed whole viral genome sequences from HuNoV isolated from P1–4 who received favipiravir treatment and from P5–6 who remained untreated (Fig. 3 and Supplementary Fig. 4). As previously shown (Fig. 1 and Supplementary Fig. 1), P1, P2 and P4 but not P3 acquired widespread favipiravir-induced sequence changes. Waterfall plots for P1 and P2 viral sequences (Fig. 3) [20] revealed, for both patients, two closely related stable clones which appeared to have evolved within the patients and this was confirmed by haplotype reconstruction using HaROLD (data not shown) [48]. For both patients, one clone predominated (>50% frequency) during favipiravir treatment (Fig. 3 and Supplementary Fig. 5) [20]. The predominance of this clone is less evident early on during treatment in P1, with considerable diversity present (Supplementary Fig. 5.A). However, at the increased favipiravir dose, this diversity disappears, and one clone is clearly dominant. Analysis of favipiravir-selected clones in P1 and P2 showed genome- wide accumulation of sequence changes, including in the RdRp, the target of favipiravir binding (Fig. 3 and Supplementary Fig. 5). In both patients, cessation of antiviral treatment reduced the abundance of the favipiravir-associated clone (Fig. 3 and Supplementary Fig. 5). In contrast, analysis of P3, in whom HuNoV sequencing was not suggestive of favipiravir-induced mutagenesis (Supplementary Fig. 1.B), did not show RdRp sequence changes (Supplementary Fig. 4.A). Patient P4, despite only receiving treatment for <3 months, did have increased numbers of favipiravir- associated sequence changes (Supplementary Fig. 1.C) but few changes at consensus level and none in the RdRp (Supplementary Fig. 4.B). Untreated patients P5 and P6 acquired few sequence changes over time (Supplementary Figs. 1.D-E and 4.C-D).

**Fig. 4:**
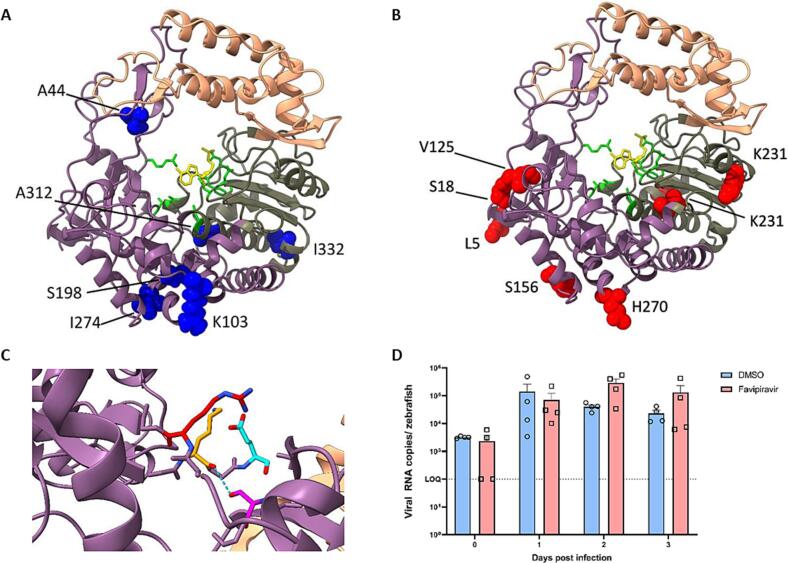
Putatively favipiravir-resistant HuNoV clones become dominant during treatment: A-B. Mapping of consensus AA changes emerging during favipiravir treatment in P1 (A. favipiravir- associated AA changes are labeled and indicated in blue) and P2 (B. favipiravir-associated AA changes are labeled and indicated in red) onto the predicted HuNoV RdRp all atom (alphafold) crystal structure. The finger domain is shown in dark pink, the palm domain in dark green and the thumb domain in peach. C. 1SH0 crystal structure showing key residues in the RdRp catalytic site (R182, K166 and E368). A44S (pink) is predicted to form a moderately strong hydrogen bond (3.368 Å) [51] with K166 (yellow) in the catalytic site thus displacing the favipiravir-contacting R182 (cyan). D. Favipiravir cannot reduce HuNoV GII.4 Sydney[P16] replication in zebrafish larvae using the post-treatment SS F for infections. Bars represent viral RNA copies/zebrafish, quantified by RT-qPCR. The dotted line represents the limit of quantification (LOQ). In every independent experiment (n = 4), 10 zebrafish larvae were harvested at each time point. Mean values ± SEM are presented, Mann-Whitney U test was used. (For interpretation of the references to colour in this figure legend, the reader is referred to the web version of this article.)

To better understand their significance, we mapped the RdRp amino acid (AA) changes present in the genotypes predominating during favipiravir treatment in patients P1 and P2 (Supplementary Table 1) onto a RdRp protein structure model (Fig. 4). Four positions (K103R, S198A, I274T, I332V) in P1 HuNoV RdRp (Fig. 4.A and in orange in Supplementary Fig. 5.A) became dominant during the first period of low dose favipiravir treatment and reduced following treatment cessation, suggesting they may possibly contribute to a relative favipiravir-resistance. All four positions showed variability among 1000 randomly selected Genbank sequences and in all cases the substituted AA had been previously observed (Supplementary Table 1). A further two P1 AA changes found during the initial treatment period, A44S and A312T (Fig. 4.A and in orange in Supplementary Fig. 5.A), became dominant when higher dose favipiravir treatment was started. The former was absent in 1000 randomly sampled Genbank sequences and the latter present in only a small proportion (1.4%) (Supplementary Table 1). Two other AA changes, I193V and L259M, which had appeared during low-dose favipiravir treatment, only rose to dominance at 6 months after the initiation of the high-dose favipiravir (shown in red in Supplementary Fig. 5.A). Both AA positions are variable (Supplementary Table 1). The persistence of I193V and L259M at low levels during low-dose favipiravir treatment and their rise to high frequency six months into treatment with high-dose favipiravir (Supplementary Fig. 5.A) rather than after introduction of treatment make it unlikely that they contributed to a favipiravir-resistant phenotype. Instead, it is possible that they are neutral or that they represent compensatory changes. A seventh AA change, N427S (shown in grey in Supplementary Fig. 5.A), became dominant in the population before the second treatment period started and may therefore either have been neutral or also have acted to stabilise putative resistance-inducing changes. In P2, eight NS sequence changes (L5K, S18N, V125M, S156N, V215I, K231R, J270N and T360S) in the HuNoV RdRp (Fig. 3.B and Fig. 4.B) also rose to high frequency during favipiravir treatment, falling again when treatment was interrupted (shown in orange in Supplementary Fig. 5.B). While all eight changes in P2 occurred in variable sites, for four (S18N, S156N, H270N and T360S) the substituted AA had not previously been observed in Genbank sequences (Supplementary Table 1).

None of the favipiravir-induced RdRp changes in P1 or P2 mapped to homologues of residues known to be associated with favipiravir resistance in influenza and chikungunya [5,49]. Five of the favipiravir- induced RdRp changes (K103R, S198A, I274T in P1; S156N, H270N in P3) appear to cluster within the same region on the protein structure (Fig. 4.A-B). No known RdRp functions are associated with this region, and none of the AA changes, alone or in combination, could be predicted in silico to change favipiravir binding or function. By simulating the random distribution of sequence changes, a thousand times, we found a significant clustering of these five mutations in the same space (p ≈ 0.001), suggesting a possibility that changes in this region may be related to the observed relative favipiravir resistance. Variant A44S in P1 (pink) is located close to both the catalytic residue R182 (6.7 Å) (cyan) and residue K166 (6.1 Å) (yellow) (Fig. 4.C), the HuNoV homologue of the known favipiravir resistant sequence change K229R in influenza [50]. In silico, the A44S change is predicted to introduce a new hydrogen bond with the amide backbone of K166 (Fig. 4.C). The estimated hydrogen bond distance of 3.269 Å has been designated a moderate to weak interaction (2.5–3.2 Å as “moderate, mostly electrostatic”, 3.2–4.0 Å as “weak, electrostatic”) [51] even without implementing any steps to refine the structure as a result of the new interaction. The finding can be replicated using the existing protein database (PDB) RdRp crystal structure 4LQ3, generating a similar distance of 3.238 Å, providing further evidence. The S44/K166 interaction could potentially cause resistance through displacing K166 with which it tightly contacts, thus effecting an indirect change in the favipiravir-contacting R182. A similar mode of action has been proposed for K229R-mediated favipiravir resistance in influenza [50].

### 3.7 Loss of viral fitness outcompetes viral selection during favipiravir treatment

In order to confirm that favipiravir-induced loss of viral fitness can truly be inferred from our findings in the zebrafish larvae (Fig. 2), we assessed whether the viruses assayed in the zebrafish larvae were representative of the original P1 samples. To do so, we compared the HuNoV sequences from SS A-D and SS F with those from viruses recovered from zebrafish inoculated with ∼1000 genome copies of each sample (as above) and harvested at day 3 pi. SS E could not be recovered from the zebrafish in sufficient quantity to sequence. Viruses recovered from both untreated and favipiravir-treated zebrafish maintained a similar pattern of drug-associated sequence changes over time, with sample A (pre-treatment) showing the lowest number of A to G and T to C changes and samples B, C and D showing increasing levels of signature changes associated with favipiravir treatment (Supplementary Fig. 6.A). The depth of sequence obtained for sample SS F was insufficient to calculate A to G and U to C changes (mean read depth < 50). Additionally, the viral RdRp sequences for SS A, B, D and F recovered from favipiravir-treated zebrafish were identical to those of their cognate P1 HuNoV RdRp sequence (Supplementary Fig. 6.B) with samples D and F showing evidence of the K103R, S198A, I274T, I332V, A312T and A44S AA changes present at the RdRp consensus level in favipiravir-treated samples. SS C from favipiravir-treated zebrafish larvae acquired one new non-synonymous change (A198) in the RdRp (Supplementary Fig. 6.B); which was present at high frequency but was not located near to the favipiravir binding site or to previously predicted favipiravir-resistance changes. Viruses recovered from untreated zebrafish larvae (—N) inoculated with samples SS A-D all acquired more genome-wide sequence changes than seen in viruses recovered from the treated zebrafish larvae (—Y) (Supplementary Fig. 6.B). New sequence changes observed in both untreated and favipiravir treated zebrafish compared with the cognate patient sample (Supplementary Fig. 6.C) could either represent a founder effect or adaptation of the virus transferred from humans into the zebrafish host. When infecting zebrafish larvae with stool sample SS F carrying the A44S change, which protein structural studies suggest potentially confers resistance to favipiravir, no reduction in viral load was observed when treated with 25 ng of favipiravir (Fig. 4.D).

Taken together, the results confirm that the specific favipiravir-induced mutagenesis observed in P1 was maintained when the virus was inoculated into zebrafish larvae and importantly, the viral RdRp sequences in favipiravir-treated zebrafish were identical or closely related to those taken from P1. Finally, we confirmed favipiravir-resistance in SS F, collected more than one year after treatment initiation, in which we previously demonstrated loss of infectivity.

## 4 Discussion

We report treatment outcomes in immunocompromised patients chronically infected with HuNoV receiving the mutagenic antiviral favipiravir, a RdRp inhibitor, in combination with nitazoxanide (in patients P1 and P4) or alone (in P1, P2 and P3). Clinical improvement, which variably included weight gain (Fig. 1 and Supplementary Fig. 1), reduction in stool frequency and improved enteral nutrition with successful weaning of parenteral nutrition [20], was sufficient to enable curative interventions, such as thymus transplantation and HSCT in patients P1 and P3. These results contrast with the use of favipiravir in other clinical settings with clinical trials against influenza, ebola and SARS- CoV-2 failing to show significant benefit even when the drug is started early in infection [9,12,52]. In 66 patients with ebolavirus who received favipiravir, low drug levels in the blood have been mooted to underlie treatment failure [21]. Similarly, pharmacological monitoring in P1 demonstrated likely inadequate blood levels during the first treatment episode where a lower favipiravir dose was given (Table 2). Doubling of the dose resulted both in higher blood levels and marked clinical improvement, although not viral clearance. Clinical improvement was also associated with adequate drug levels in P4, with a decrease in VLs, but no clearance either. Favipiravir plasma levels were not measured in P2 and P3. P3's initial higher dose resulted in a rapid fall in VLs (Supplementary Fig. 1), although this was associated with toxicity necessitating treatment interruption. Re-starting favipiravir at a lower dose maintained clinical improvement even though this was no longer associated with falling VLs.

Under the clinically tolerated favipiravir dose regimens, changes in VLs, if any, were minimal (Fig. 1 and Supplementary Fig. 1). Viral deep sequencing however demonstrates that clinical improvement coincided with the accumulation of A to G and T to C sequence changes through favipiravir-induced polymerase mispriming (Fig. 1 and Supplementary Fig. 1) [20,53]. Although enzymatic studies pointed out that favipiravir can act ambiguously as a guanosine and adenine analogue leading to the inhibition of HuNoV RNA synthesis, we noted a more pronounced role as adenosine analogue [54]. These signature sequence changes appear to be dose-dependent, increasing with higher favipiravir plasma concentrations, and decreasing with treatment interruptions, in P1, P2 and P4 (Fig. 1 and Supplementary Fig. 1) [20]. The absence of signature sequence changes in P3, together with the rapid VL falls following the start of high-dose favipiravir treatment, suggests that rather than cause mispriming though incorporation into the RNA genome, high-dose favipiravir may in this case have acted by directly inhibiting the HuNoV polymerase. A similar effect has been noted for influenza treated in vitro with high doses of favipiravir [8]. No data on mutagenesis is available for P3 HuNoV at the lower dose, but our findings in P1/2/4 mirror those found after favipiravir treatment in a hamster model of SARS-CoV-2 infection, where a reduction in viral-associated lung pathology was associated in a dose-dependent manner with increasing drug-driven mutagenesis [7].

Favipiravir has also been shown to reduce the viral infectivity of several RNA viruses both in vitro and in vivo, including influenza, lassa fever, ebola and chikungunya [11], in some cases in association with demonstrable drug mutagenesis [8,40]. Similar findings for HuNoV have been precluded by the difficulties in its propagation in vitro or in animal models which have also proved a barrier to the investigation of HuNoV pathophysiology in general and to the validation of other potential therapeutic agents. The zebrafish model we recently established is well suited to evaluate drug efficacy and HuNoV infectivity, since it requires an inoculum of only ∼10^3^ viral RNA copies for infection to occur, with HuNoV showing a tropism for the intestine after inoculation in the yolk (their food reservoir) thereby mimicking the oral route [29,41]. We have previously shown that we could culture HuNoV samples obtained from chronically infected individuals in zebrafish larvae [29], unlike what has been reported for the human intestinal enteroid model thus far [26,55]. Exploiting the availability of this new animal model as well as the access to longitudinally stored HuNoV positive stool samples of patient P1 collected over a treatment period of one year, we are able to show ∼1 log_10_ increase in the 50% infectious dose in HuNoV shed during treatment (Fig. 2.D). Importantly, the model allows us to demonstrate for the first time that clinical improvement in the patient and the accompanying increase in viral A to G and T to C sequence changes (Fig. 1) [20] are associated with progressive loss of HuNoV infectivity during treatment with favipiravir alone (SS E-F) or in combination with nitazoxanide (SS B—D). During treatment interruption, reduction in favipiravir-induced signature sequence changes was associated with clinical deterioration in P1, while reintroduction of treatment restored the mutagenic signature, again reduced viral infectivity, and improved the clinical picture (Fig. 1, Fig. 2) even after discontinuation of nitazoxanide (SS E-F). The exception was sample SS D which, despite apparently high numbers of transition sequence changes, was less impaired for replication. Sample SS D differed from sample SS C by having acquired the putative drug resistant A44S change and other AA changes in the RdRp, including A312T and P35S, which appeared prior to restarting treatment (Supplementary Fig. 5.A). All three AA changes were also shared with samples SS E and SS F, which unlike sample SS D, did show the expected loss of infectivity (Fig. 2). Sample SS E and SS F had also acquired the RdRp changes I193V and L259M (shown in red, Supplementary Fig. 5.A). While it is not clear why SS D regained infectivity, it is possible that this resulted from the first three acquired RdRp AA changes. These may have partially restored RdRp function to P1 norovirus during testing for infectivity in the absence of drug. Only for RdRp function to be lost again after acquisition of I193V and L259M in SS E and SS F during favipiravir treatment, which instead may have acted to stabilise RdRp in the presence of drug but reduced fitness when virus was cultured without drug in zebrafish for fitness studies. The dominant SS D strain recovered from untreated zebrafish also carried different sequence changes compared with untreated samples SS A and SS B (which were similar to one another), and SS C, including in ORF 1 where the genes involved in norovirus replication are located (Supplementary Fig. 6.C). It is possible that the different sequence changes acquired by untreated zebrafish sample SS D also contributed to its apparent partial recovery of replication fitness by comparison to samples SS B and SS C. In contrast, there were few new changes present in sample SS F recovered from untreated zebrafish, the sequence of which most closely resembled the original patient SS F sequence (Supplementary Fig. 6.C), and which replicated worse (with the highest ID_50_) in zebrafish than all the other samples. Overall, the in vivo experiments demonstrate a loss of HuNoV infectivity over time, coinciding with accumulation of sequence changes and persisting months after nitazoxanide discontinuation, suggesting that favipiravir-induced mutagenesis may contribute the most to treatment success. Nitazoxanide may have played a role by boosting the innate immune response early after infection (Fig. 2), thereby potentiating the action of favipiravir and promoting dose-sparing synergy.

Taken together, the data from this case series represent the first evidence that favipiravir and nitazoxanide can be used to treat chronic norovirus infection in immunocompromised patients, a condition for which there are currently no licensed or unlicensed treatments and no candidate drugs in development by pharmaceutical companies. The viral sequencing data from samples collected in 4 treated and 2 untreated patients suggest there may be an association between the count of drug- induced sequence changes and the loss of infectivity over time; these preliminary results need to be confirmed in larger data sets. Upon demonstration of a true correlation, it may be possible to identify a mutational threshold associated with favipiravir efficacy, in which case the frequency of mutational signatures could be useful for monitoring the response to mutagenic RdRp inhibitors especially given the modest VL reductions.

Non-lethal drug mutagenesis predisposing to persistence of mutated viral genomes has been predicted to be a potential threat of emergence of drug resistance not only against the RdRp inhibitor in-use, but against other drugs, including therapeutic monoclonal antibodies [56]. Our unique dataset of HuNoV sequences collected longitudinally over 2–12 months from three patients in whom we documented non-lethal drug mutagenesis, provides an opportunity to examine the evolution of resistance. We show that prolonged favipiravir usage may indeed result in the selection over time of viral populations carrying AA changes in the RdRp (Fig. 3), which are not seen in untreated patients, pointing to emergence of drug resistance. The RdRp A44S mutation is a particularly strong candidate for favipiravir resistance through its proposed interaction with K166 (Fig. 4), the homologue of K229 in influenza, where change of an arginine has been demonstrated to cause favipiravir-resistance [50]. Other AA changes in P1 are less convincing but the possibility of epistatic effects contributing to the development of drug resistance remains. These may further have been enhanced by the clustering of these sequence changes (Fig. 4). Given the loss of infectivity despite the presence of possible resistance-inducing AA changes in the RdRp, these AA changes are likely to also induce impaired fitness, particularly in samples SS E and SS F (Fig. 2). We conclude that the RdRp changes although contributing to a more resistant phenotype had little or no impact on the patient, even in the presence of drug. Selection of these sequence changes by ongoing favipiravir treatment may even have contributed to the decreasing viral fitness measured in the zebrafish, thereby facilitating the observed clinical improvement and (modest) fall in HuNoV VLs in absence of functional T-cell immunity. By the same token, although drug-associated mutagenesis was observed in the HuNoV capsid, including in known epitopes, potentially allowing the virus to evade immunity, these variants are likely to be impaired for fitness and less transmissible.

This report has several limitations. The four patients described here received treatment on a compassionate basis and not as part of a randomised clinical trial, which can lead to observer bias in relation to clinical outcomes. While sample size is small, the accompanying PK and HuNoV sequencing data strongly support a drug effect in P1, P2, P3 and P4. For P1 specifically sequencing data is available for samples collected over a treatment period of more than one year. The unique zebrafish infectivity data relating to P1 further confirms this. Although we did show that mutated genomes at the end of treatment were less fit in the zebrafish (Fig. 2), the death of P1 from sepsis, unrelated to HuNoV, prevented us from testing whether these mutated viruses would have persisted in a human host when treatment was stopped, potentially posing a risk for onward transmission of resistance. In absence of paediatric pharmacokinetic data for favipiravir, patients were given variable dose regimens. Additionally, given the compassionate nature of the administered treatments, a heterogeneity in access and combinations of antivirals exists, which may have impacted clinical outcomes. The use of these drugs is not without complications. Liver function test (LFT) abnormalities were noted during favipiravir treatment of P1, P2 and P3 and nail discolouration, a known favipiravir-associated toxicity, in P3 and P4. Stopping favipiravir (and other hepatotoxic drugs) reversed the transaminitis in P1, P2 and P3 and nail discoloration in P3 and P4. Moreover, in P2 and P3, favipiravir was restarted at a lower dose with continued clinical benefit and no significant toxicity, while in P1, restarting at a higher dose also led to clinical benefit with no toxicity. Favipiravir does, however, require careful clinical and blood monitoring, and where this cannot be implemented, a decision to stop drug treatment may need to be taken despite the consequences, as happened for P4.

In summary, we demonstrate for the first time that clinical improvements in HuNoV infected patients treated with favipiravir, including in combination with nitazoxanide, are associated with loss of viral infectivity coinciding with a genome-wide accumulation of viral sequence changes. Although prolonged treatment resulted in selection of virus carrying changes that may possibly confer some degree of HuNoV resistance to favipiravir as well as potential evasion of immunity, the loss of viral fitness as measured in the zebrafish model and the absence of variants carrying putative resistance mutations in untreated zebrafish, militates against the likely onward transmission of resistance or immune escape variants. The data thus provide support for the compassionate use of favipiravir, in combination with nitazoxanide, to treat chronic HuNoV infections in immunocompromised hosts, particularly as a bridging therapy to corrective cell therapies such as HSCT and/or to recovery of cellular immunity. Without treatment these patients are often ineligible for corrective treatment due to significant morbidity, and they are at risk of early mortality. While treatment with favipiravir does require careful monitoring and optimally, measurement of drug levels, we show that toxicity can be easily reversed, and continued treatment is safe. Importantly, the development of drug resistance following treatment with mutagenic antivirals occurs only after prolonged treatment and is associated with a high fitness cost making unlikely the emergence of novel resistant or immune escape HuNoV variants capable of transmission. The broad-spectrum activity of RdRp inhibitors makes it likely that these conclusions apply to other RNA virus infections, including SARS-CoV-2.

## Funding

AYK is supported by the 10.13039/100010269Wellcome Trust [222096/Z/20/Z].

ER receives funding from 10.13039/501100003130Research Foundation Flanders (FWO) [Project Number: 11K7922N].

JP received funding from 10.13039/501100000833Rosetrees Trust.

IC is funded by the 10.13039/501100000272National Institute for Health Research (NIHR) [NIHR301575].

EH and EGD are supported by LetterOne in conjunction with GOSH Children's Charity.

The work leading to this research was supported by KU Leuven internal funds (C2 project) and a KUL starting grant to JRP (STG/21/028).

JB receives funding from the NIHR UCL/10.13039/501100012317UCLH Biomedical Research Centre.

Research at 10.13039/501100003784Great Ormond Street Hospital for Children NHS Foundation Trust is supported by the NIHR GOS/10.13039/501100001282ICH 10.13039/100014461Biomedical Research Centre.

## Author contributions

Conceptualization: JRP and JB. Methodology: AYK, ER, IC, JS, JRP and JB.

Investigation: AYK, ER, JP, IC, OC, TB, CS, JS, RG, JRP, JB.

Funding acquisition: JRP and JB.

Writing – original draft: AYK, ER, JRP and JB. Writing – review & editing: All.

## Declaration of competing interest

Authors declare that they have no competing interests.

## Data Availability

Raw fastq files have been submitted to the BioProject database of the National Center for Biotechnology Information (NCBI) (BioProject ID: PRJNA1005817). Clinical data is stored within the firewall of UK National Health Service. The data that support the findings of this study are available on request from the corresponding author.

## Appendix A Supplementary data

Supplementary material Image 1
