# Supplementary material for: Favipiravir induces HuNoV viral mutagenesis and infectivity loss with clinical improvement in immunocompromised patients
Source: Clin Immunol. 2024 Feb;259:109901. doi: 10.1016/j.clim.2024.109901 (PMC11933534; doi:10.1016/j.clim.2024.109901)
Supplement: Supplementary file 1 — Supplementary material [file mmc1.pdf]

Supplementary materials

A P2

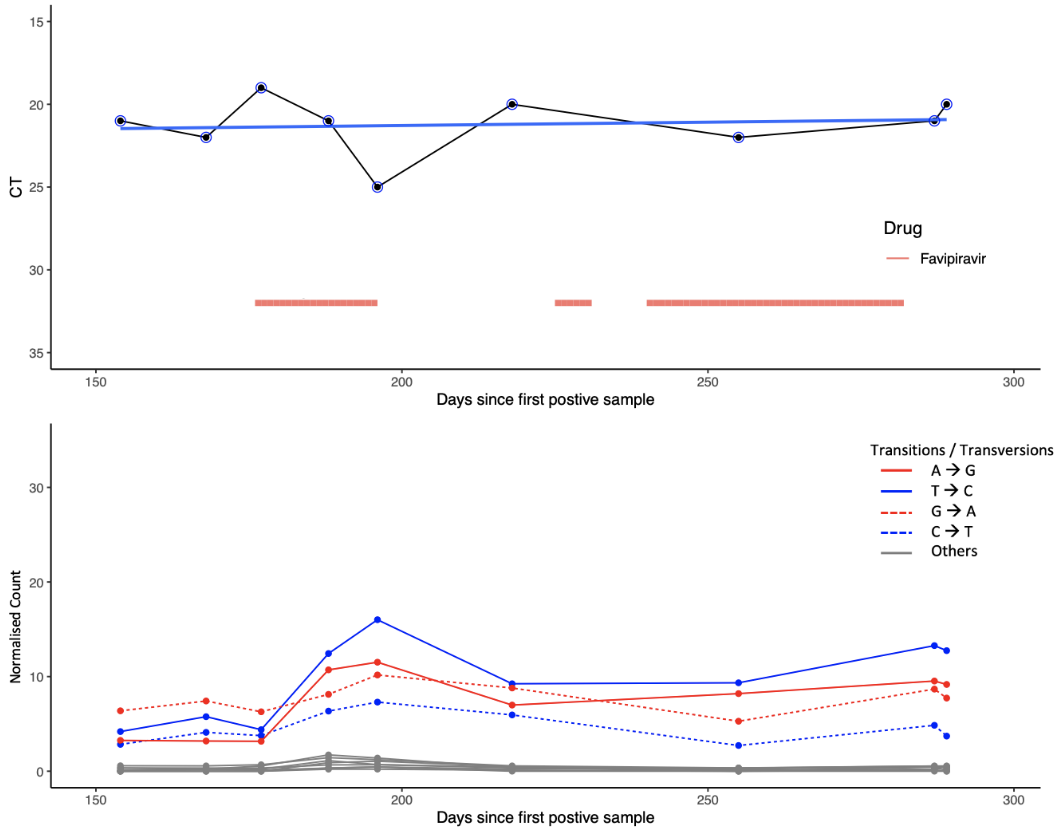

B P3

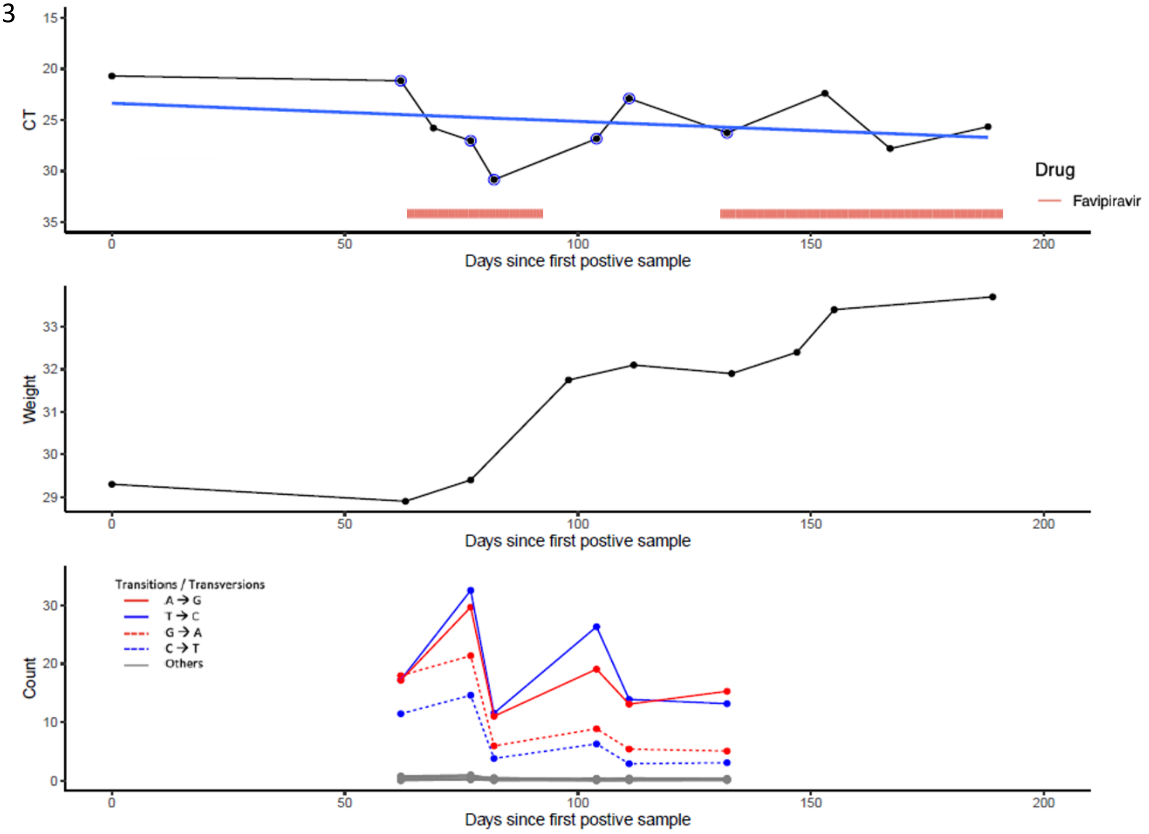

C

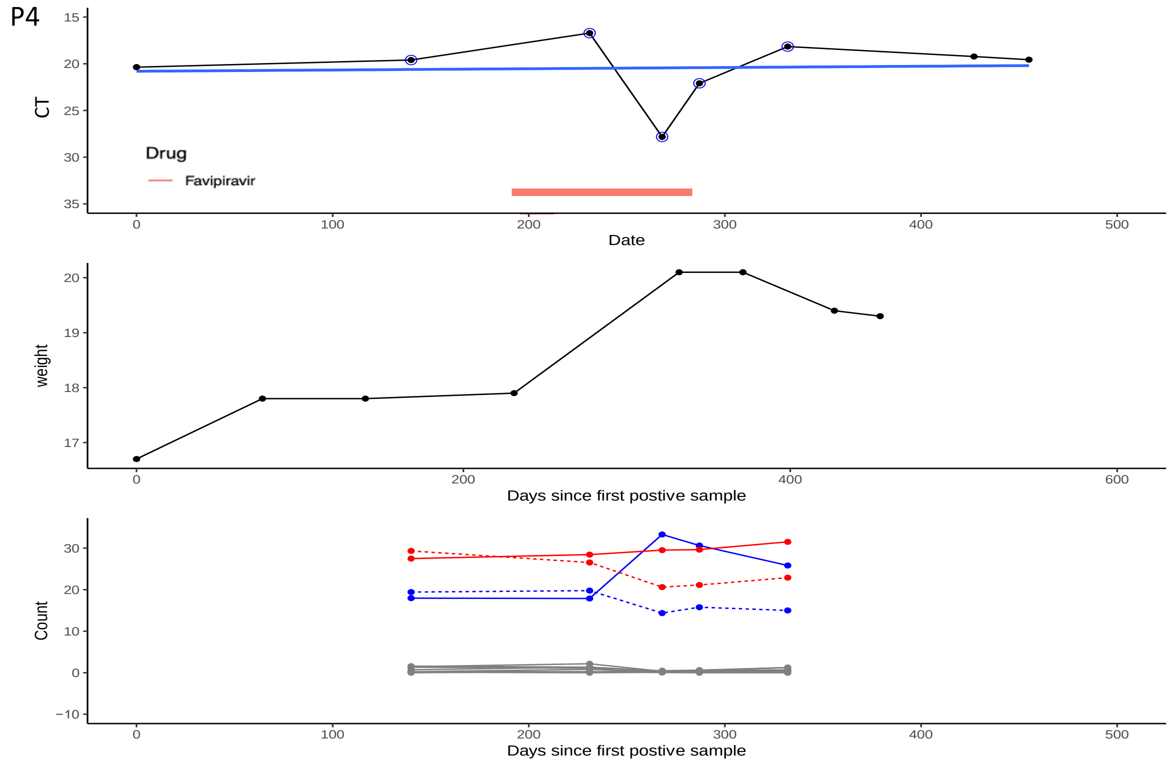
P4
CT
Drug
Favipiravir
Date
weight
Days since first postive sample
Count
Days since first postive sample

D

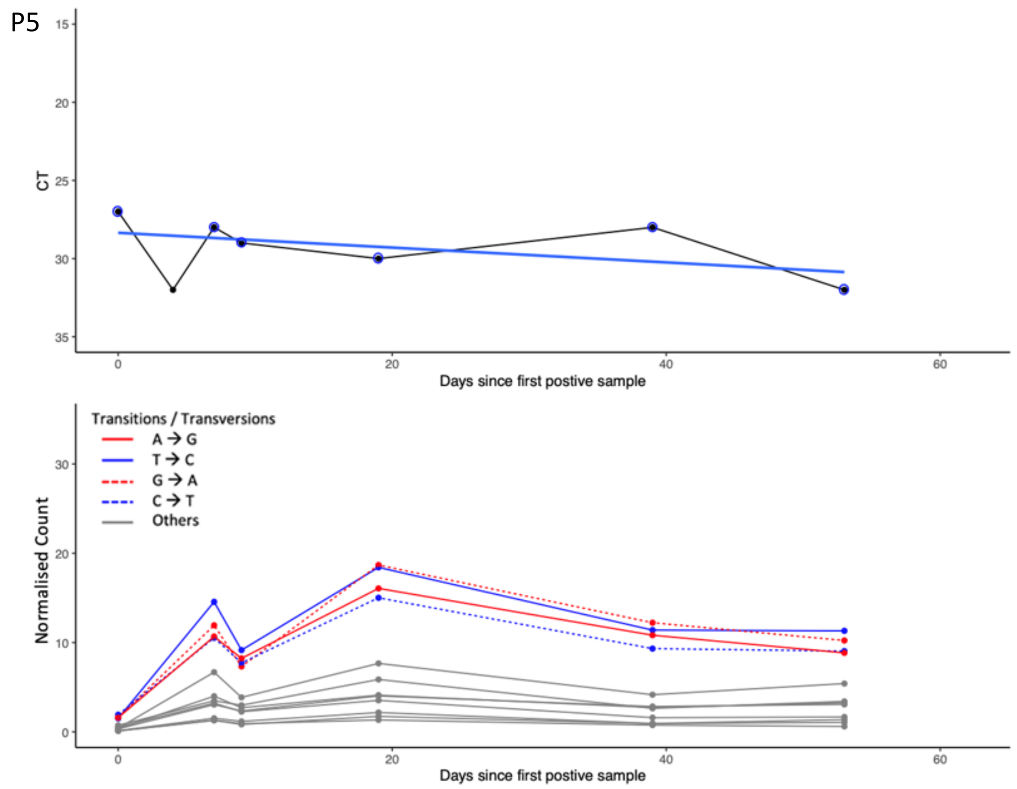
P5
CT
Days since first postive sample
Transitions / Transversions
A → G
T → C
G → A
C → T
Others
Normalised Count
Days since first postive sample

E

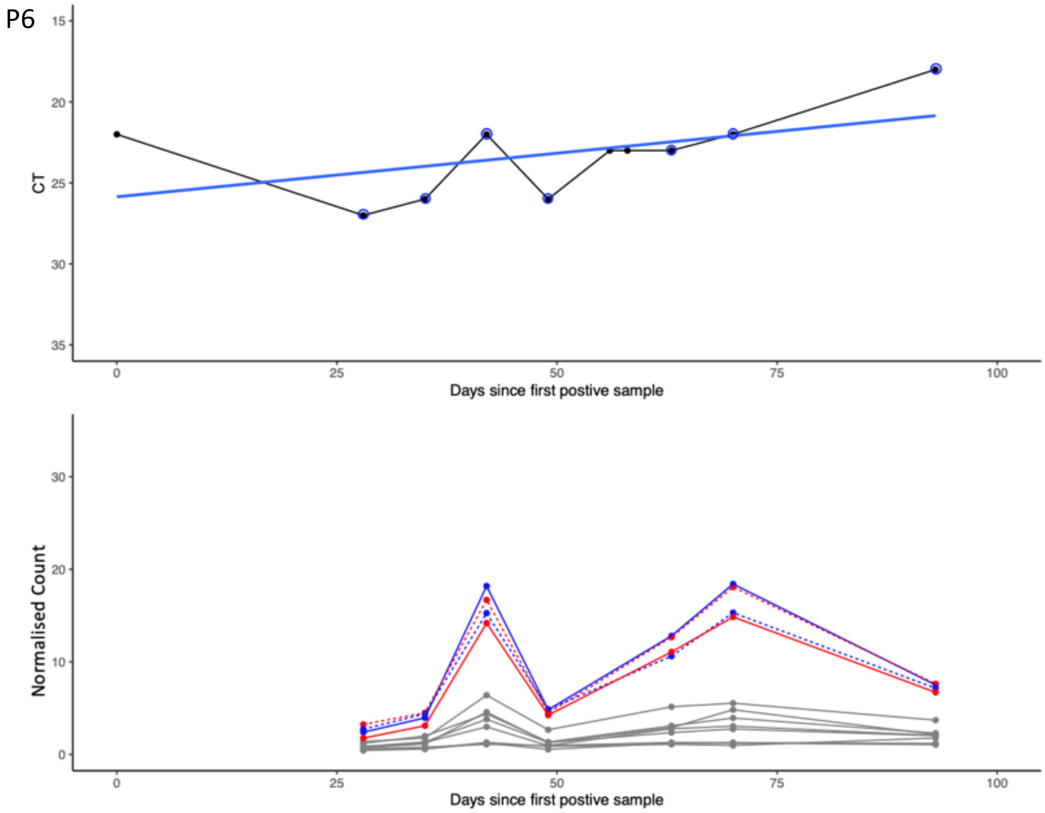

Fig. S1: Clinical and virological monitoring in treated patients P2-P4 and untreated patients P5-6: A-C. Monitoring of treatment response in patients P2-P4 over time, indicated in days since first positive stool sample. For each patient, treatment periods are indicated at the top (red line for favipiravir, green line for nitazoxanide). Samples which have been processed for viral deep sequencing are circled in blue. For each patient, the top panel shows HuNoV viral load quantification by RT-qPCR from stool samples obtained during the same period. The viral load is expressed in cycle threshold (Ct) values, with increasing Ct values corresponding to a reduction in viral load. The middle panel (P3 and P4) shows patient weight before and during antiviral treatment, as one of the clinical parameters used to monitor treatment outcomes. The bottom panel shows the counts of variants A→ G and T→C; solid lines, associated with favipiravir mutagenesis and G→A and C→T dotted lines, complementary strand mutations for each stool sample processed for viral deep sequencing before and during the periods of favipiravir treatment, normalised using Watterson's theta. Other transitions and transversions are shown in grey. **D-E.** For the untreated patients P5-6, the top panel shows HuNoV viral load quantification by RT-qPCR from stool samples obtained during follow-up while the bottom panel shows the counts of variants A→ G and T→C; solid lines, and G→A and C→T dotted lines, complementary strand mutations for each stool sample processed for viral deep sequencing, normalised using Watterson's theta.

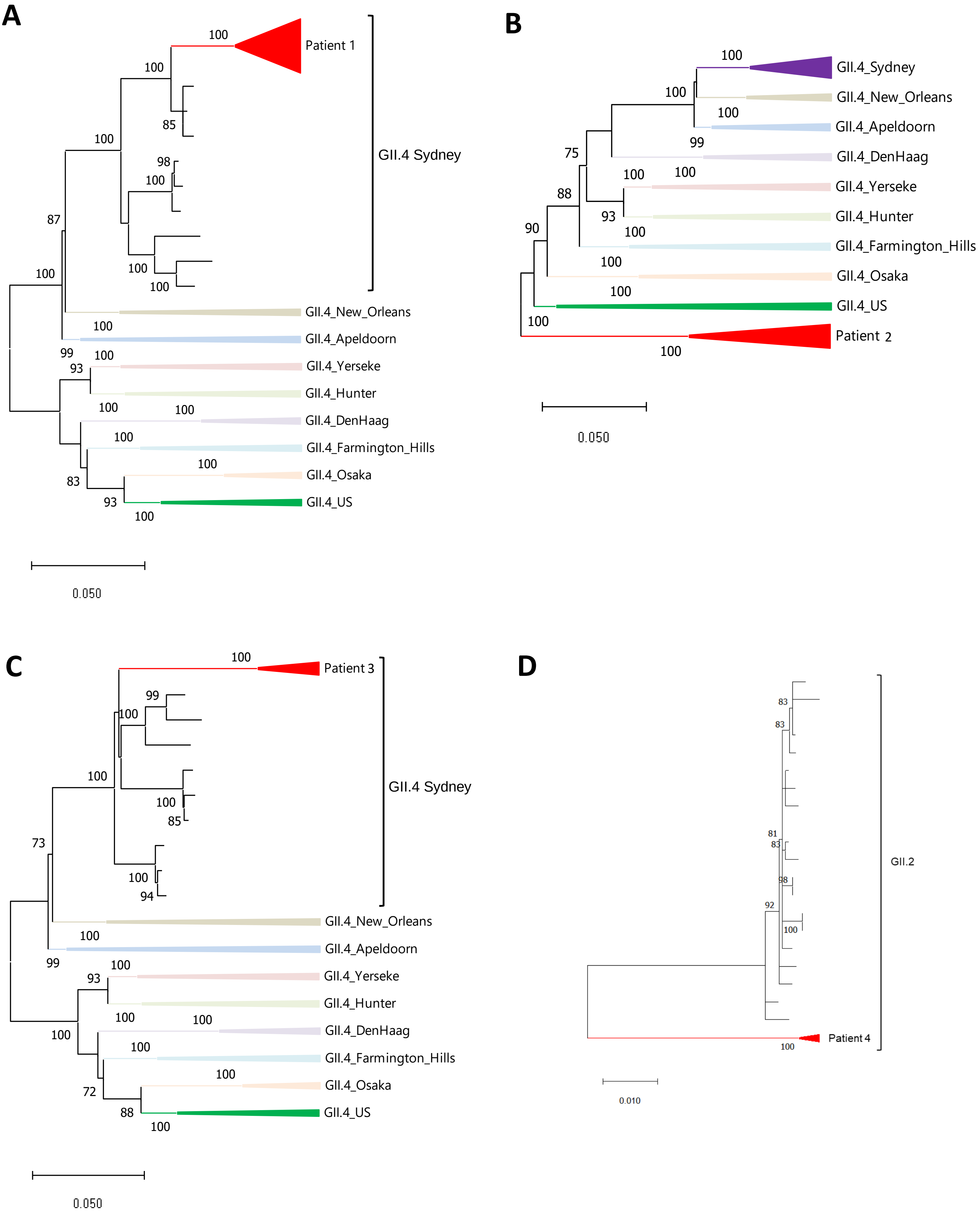
A
Patient 1
GII.4 Sydney
GII.4_New_Orleans
GII.4_Apeldoorn
GII.4_Yerseke
GII.4_Hunter
GII.4_DenHaag
GII.4_Farmington_Hills
GII.4_Osaka
GII.4_US
0.050
B
GII.4_Sydney
GII.4_New_Orleans
GII.4_Apeldoorn
GII.4_DenHaag
GII.4_Yerseke
GII.4_Hunter
GII.4_Farmington_Hills
GII.4_Osaka
GII.4_US
Patient 2
0.050
C
Patient 3
GII.4 Sydney
GII.4_New_Orleans
GII.4_Apeldoorn
GII.4_Yerseke
GII.4_Hunter
GII.4_DenHaag
GII.4_Farmington_Hills
GII.4_Osaka
GII.4_US
0.050
D
GII.2
Patient 4
0.010

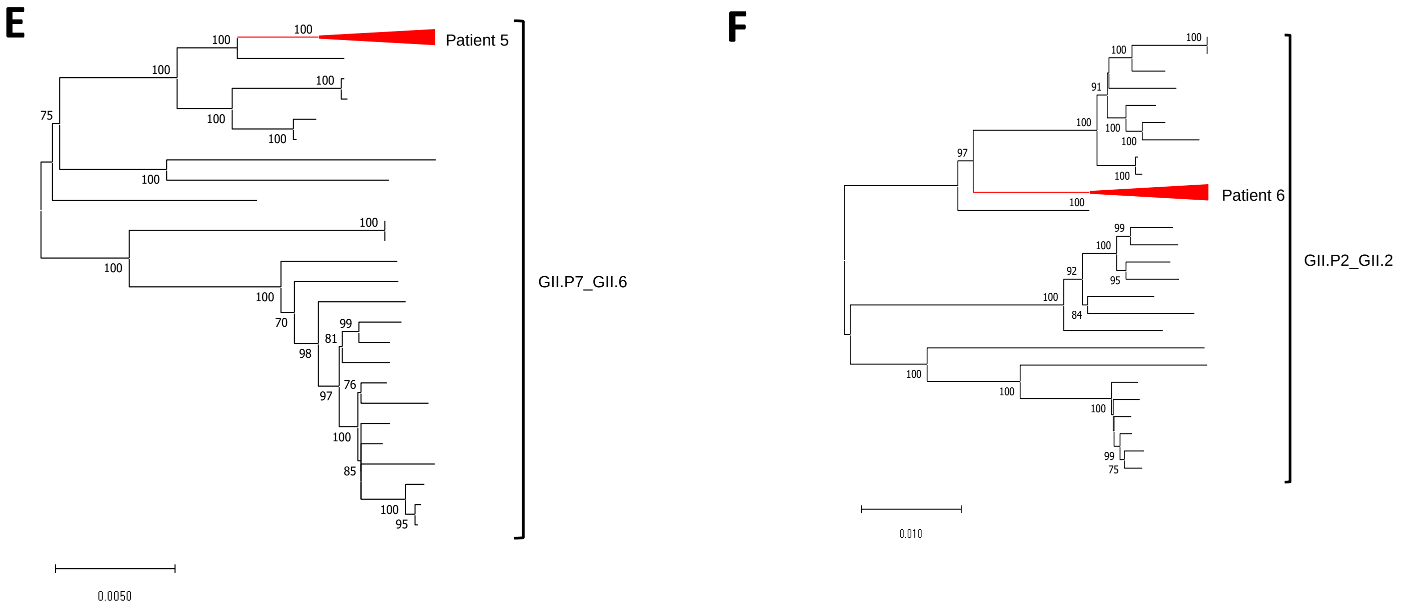

Fig. S2: Viral genotyping and phylogenetic analysis: Maximum likelihood phylogenetic analysis in relation to representative sequences from previous norovirus pandemics and epidemics are show for **A.** Patient 1, **B.** Patient 2, **C.** Patient 3, **D.** Patient 4, **E.** Patient 5, and **F**. Patient 6. Closely related sequences are collapsed into fans. Scale bars indicate the changes/site.

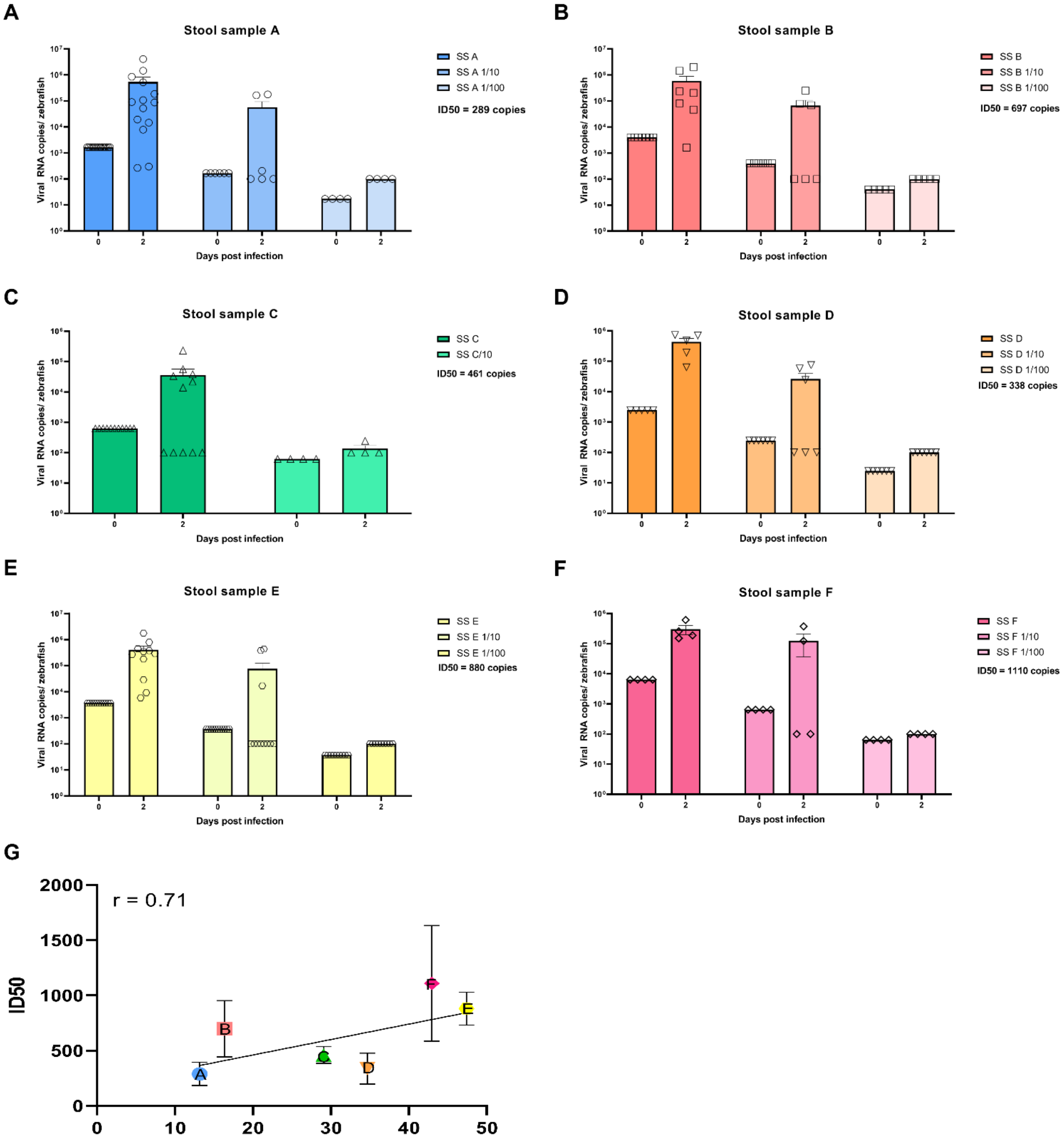

Fig. S3: Quantifying the ID50 using serial dilutions of P1 stool samples. A-F. Serial dilutions of each stool sample (SS A-F) used for ID_{50} quantification using the Reed-Muench method. For all graphs: Bars represent viral RNA copies/zebrafish, quantified by RT-qPCR. In every independent experiment (n=2-14), 10 zebrafish larvae were harvested at each time point. Mean values ± SEM are presented. **G.** The correlation between the ID50 of all stool samples (SS A-F) and their normalized polymorphism count (both A→ G and T→C) is displayed. The Spearman correlation coefficient was calculated (r = 0.71). Mean values ± SEM are presented.

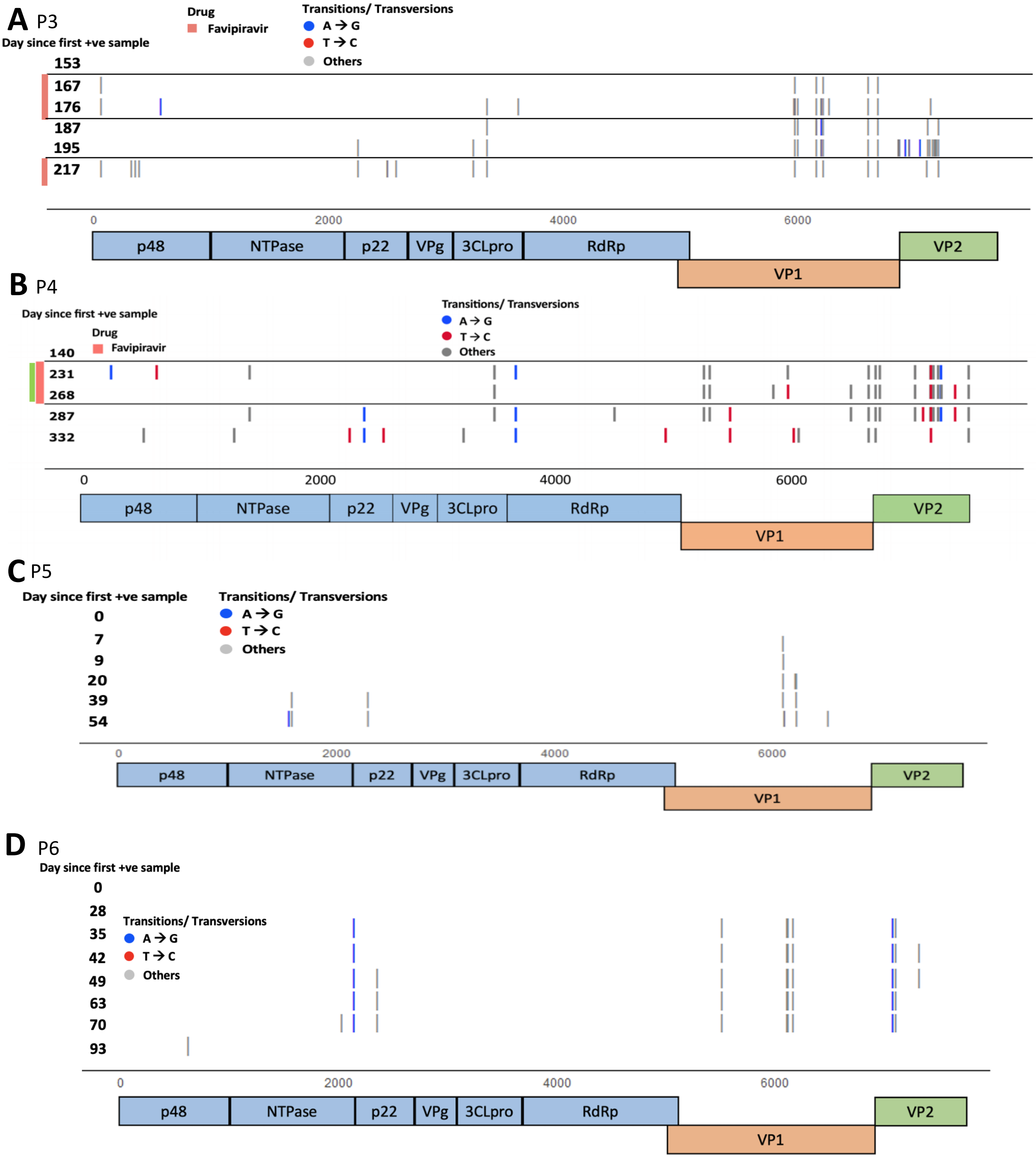

Fig. S4: Nonsynonymous HuNoV sequence changes occurring over time in treated patients P3-4 and untreated patients P5-6. Waterfall plots showing nonsynonymous sequence changes at consensus level (>50%) across the norovirus genome for (**A-B.**) treated patients P3-P4 and (**C-D.**) untreated patients P5-P6. Changes are compared to the baseline patient reference sample. X-axis = positions across the norovirus genome, Y-axis (each row) indicates each sample. Vertical bars on the left indicate whether the sample was taken during treatment. Red = Favipiravir, green = nitazoxanide. Sequence changes likely to be related to favipiravir treatment are shown in blue (A to G) and red (T to C). Consensus changes due to other nucleotide transitions and transversions are shown in grey.

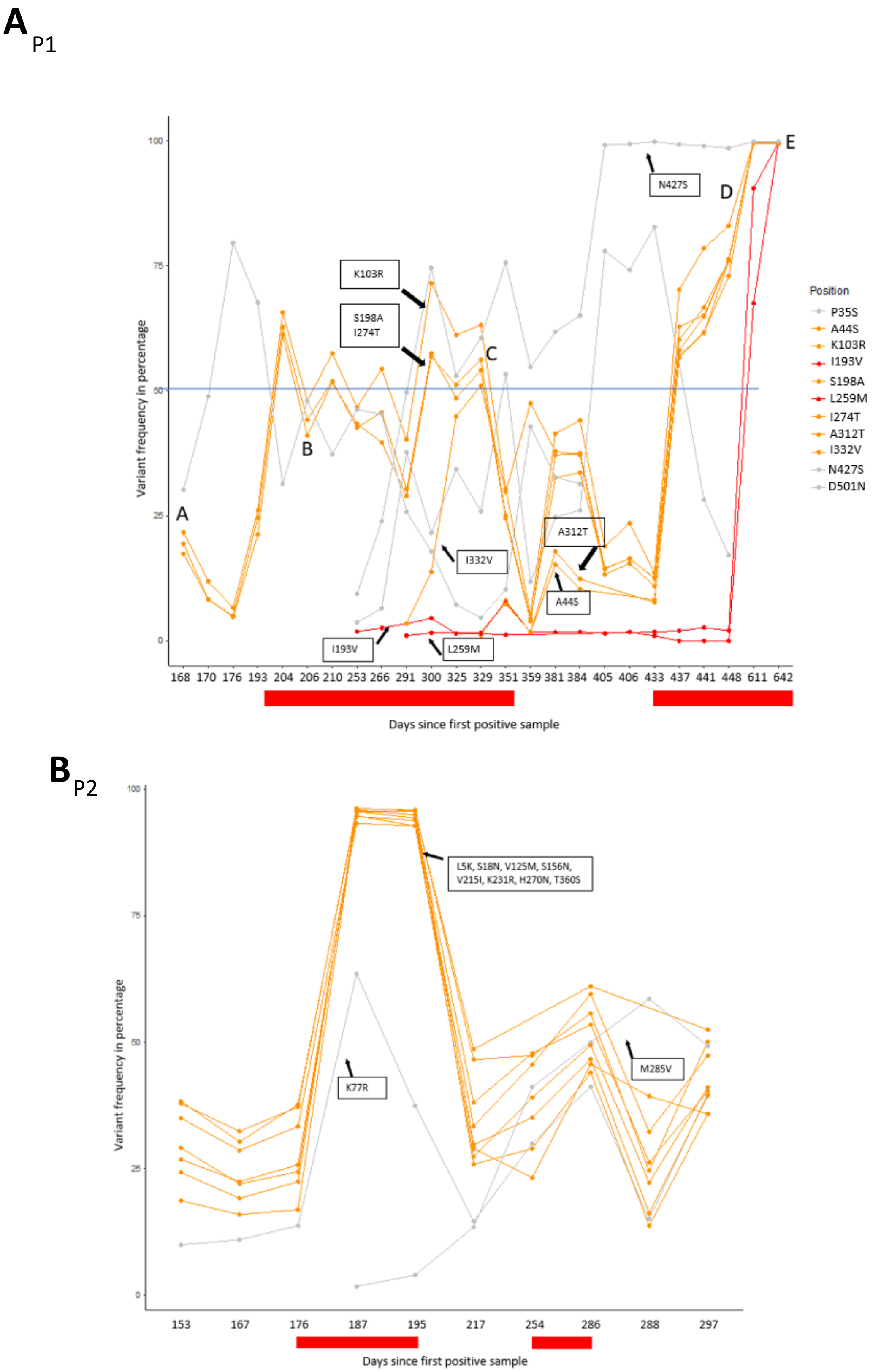

Fig. S5: Allele frequencies for RdRp non synonymous (NS) nucleotides is increased by favipiravir treatment: Allele frequencies **A.** in P1 and **B.** in P2 for NS nucleotides in the HuNoV RNA dependent RNA polymerase gene. Those NS variants which rise to >50% frequency with favipiravir treatment are labelled and shown in orange. NS nucleotides with changes in allele frequencies not associated with drug treatment are shown in grey. Periods of favipiravir treatment are represented by red bars at the bottom. Two nucleotides rising to high frequency in P1 several months after the second period of treatment are shown in red. The X-axis indicates when sample was collected but does not represent a linear scale for time.

A

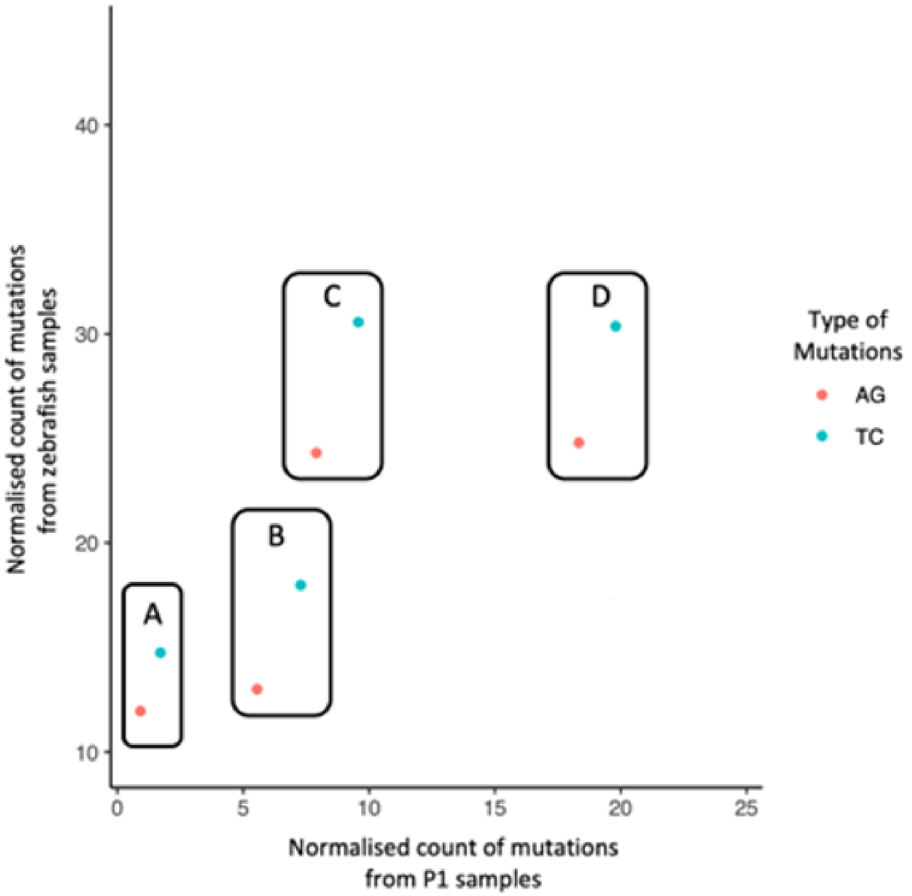

B

	35	44	76	103	193	198	259	274	312	332	427	502
SampleA	P	A	K	K	I	S	L	I	A	I	N	D
SampleA-N	.	.	.	.	.	.	.	.	.	.	.	N
SampleA-Y	.	.	.	.	.	.	.	.	.	.	.	.
SampleB	.	.	.	.	.	.	.	.	.	.	.	N
SampleB-N	.	.	.	.	.	.	.	.	.	.	.	D
SampleB-Y	.	.	.	.	.	.	.	.	.	.	.	N
SampleC	S	.	.	.	.	.	.	.	.	.	S	.
SampleC-N	P	.	.	R	.	A	.	T	T	.	S	.
SampleC-Y	S	.	.	.	.	A	.	.	.	.	S	.
SampleD	.	S	.	R	.	A	.	T	T	V	S	.
SampleD-N	.	A	R	R	.	A	.	T	T	I	S	.
SampleD-Y	.	S	.	R	.	A	.	T	T	V	S	.
SampleF	.	S	.	R	V	A	M	T	T	V	S	.
SampleF-N	.	S	.	R	V	A	M	T	T	V	S	.
SampleF-Y	.	S	.	R	V	A	M	T	T	V	S	.

C

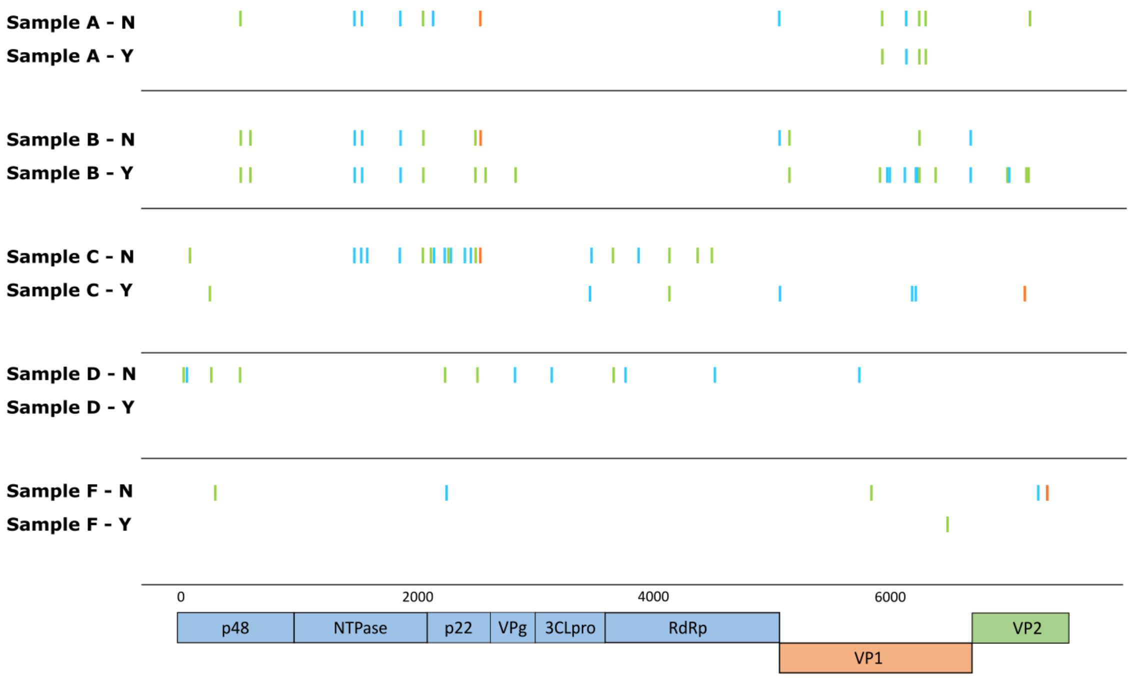

Fig. S6: Norovirus sequences for samples SS A-D from untreated (N) and favipiravir-treated (Y) zebrafish as compared with the sequence of the original sample obtained from the patient: A. Numbers of normalised A to G and T/U to C sequence changes for samples with mean read depth >100 recovered from treated zebrafish i.e. SS A-D. **B.** Detailed listing of amino acid changes in the RdRp of samples SS A to D and F, and their cognate samples from untreated (SS A to D and F-N) and favipiravir-treated (SS A to D and F-Y) zebrafish. The HuNoV amino acid sequence for each sample is shown followed by the untreated and treated zebrafish sequences. Changes in SS B, C and D HuNoV amino acid sequences compared with the baseline SS A sequence are shaded orange. Changes in zebrafish sequences compared with the cognate HuNoV SS sequence are shaded grey. AA sequences identical to the baseline SS A HuNoV sequence are left blank. Zebrafish AA sequences that are identical to the cognate HuNoV sequence but different from baseline SS A HuNoV sequence are shown as white with the AA labelled. **C.** Genome wide waterfall plots showing consensus amino acid changes across the norovirus genome (bottom) for the untreated (N) and favipiravir treated (Y) zebrafish compared to the original patient samples SS A-D and SS-F. Blue A to G, orange T to C and green other.

Table S1: Frequency in 1000 Genbank sequences of non synonymous RdRp substitutions observed with favipiravir treatment in P1 and P2:

P1

Amino Acid Changes	Nucleotide Changes	Original AA	Mutated AA	Others	Stop Codon	NA
A44S	G-->T	992	0	0	0	8
K103R	A-->G	923	30	5	42	0
I193V	A-->G	892	108	0	0	0
S198A	T-->G	588	412	0	0	0
L259M	T-->A	954	46	0	0	0
I274T	T-->C	735	173	92	0	0
A312T	G-->A	928	14	58	0	0
I332V	A-->G	812	188	0	0	0

P2

L5K	T-->A	0	993	4	3	0
S18N	G-->A	93	0	603	304	0
V125M	G-->A	721	159	120	0	0
S156N	G-->A	7	0	808	185	0
V215I	G-->A	725	275	0	0	0
K231R	A-->G	968	32	0	0	0
H270N	C-->A	216	0	783	1	0
T360S	A-->T	174	0	826	0	0

Drug-associated mutations are highlighted in red (T→C) and in blue (A→G).
